# Comparative efficacy and safety of targeted therapeutics or immunotherapy agents combined with chemotherapy as first-line treatment for advanced biliary tract cancer: a systematic review and network meta-analysis

**DOI:** 10.1186/s12885-025-15370-8

**Published:** 2025-12-03

**Authors:** Haodong Ma, Zixuan Wang, Yingying Tong, Haojie Li, Yanmiao Han, Dezhi He

**Affiliations:** 1 Department of Gastroenterology, The First Affiliated Hospital of Zhengzhou University, Zhengzhou, Henan 450052 China; 2 Department of Gynecology, The First Affiliated Hospital of Zhengzhou University, Zhengzhou, Henan 450052 China

**Keywords:** Network meta-analysis, Advanced biliary tract cancer, Immunotherapy, Targeted therapy

## Abstract

**Background:**

Although durvalumab or pembrolizumab combined with gemcitabine plus cisplatin (GC) remains the standard first-line therapy, novel targeted agents and immunotherapies integrated with chemotherapy have demonstrated promising efficacy. However, the crosswise comparison between each regimen is rare. Therefore, we comparative the efficacy and safety of targeted therapeutics or immunotherapy agents combined with chemotherapy as first-line treatment for advanced biliary tract cancer.

**Methods:**

We included 18 randomized controlled trials (RCTs) meeting selection criteria to evaluate first-line targeted/immunotherapy-chemotherapy combinations for advanced BTC. Among them, 15 RCTs were analyzed via Bayesian network meta-analysis (NMA) for the average group, while 6 RCTs were used for pairwise meta-analyses of molecular subgroups, with three studies contributing to both analyses. Outcomes included overall survival (OS), progression-free survival (PFS), objective response rate (ORR), and serious adverse events (SAEs). Subgroup analyses were performed for KRAS wild-type and PD-L1-positive populations.

**Results:**

Durvalumab (HR 0.76, 95% CI 0.64–0.91) and pembrolizumab (HR 0.83, 95% CI 0.72–0.95) combined with GC significantly improved OS versus chemotherapy alone, with comparable SAE rates. Sintilimab plus anlotinib and GC achieved the best PFS (HR 0.47, 95% CI 0.28–0.80), though OS benefits were nonsignificant. EGFR inhibitors prolonged PFS in KRAS wild-type patients (HR 0.72, 95% CI 0.52–0.98). Bintrafusp alfa and cetuximab showed higher SAE risks (OR 2.26 and 1.95, respectively).

**Conclusion:**

Our findings directly inform clinical guidelines, address gaps in current therapeutic decision-making. Durvalumab or pembrolizumab combined with GC are optimal first-line regimens for advanced BTC, balancing survival benefits and safety. Sintilimab plus anlotinib combined with GC demonstrates superior PFS but requires further validation. While EGFR inhibitors plus chemotherapy demonstrate potential in KRAS wild-type patients, confirmation in large-scale RCTs is required. PD-L1 expression may represent a promising predictive biomarker for response to PD-1 inhibitor therapy.

**Supplementary Information:**

The online version contains supplementary material available at 10.1186/s12885-025-15370-8.

## Introduction

Biliary tract cancer (BTC) is a group of rare and aggressive malignancy, encompassing intrahepatic or extrahepatic cholangiocarcinoma, gallbladder cancer, or ampullary cancer [1]. BTC accounts for less than 1% of all cancers, with significant geographic variation in incidence [2]. The disease is more prevalent in the Asia-Pacific region and South America, while its incidence remains lower in Europe and North America [1].Over recent decades, the global incidence of BTC has risen, continuing to pose a substantial health burden worldwide [3].

BTC is often asymptomatic or presents with nonspecific symptoms in its early stages, leading to late-stage diagnoses. Approximately 70% of patients are diagnosed when the disease is already metastatic or unresectable [4–6]. Even after radical surgery, recurrence is common among patients with early-stage disease [7]. For those with advanced or unresectable BTC, systemic chemotherapy remains the primary treatment option. The phase III ABC-02 trial established the combination of gemcitabine and cisplatin as the standard first-line chemotherapy for advanced BTC [8]. Additionally, gemcitabine combined with oxaliplatin has shown efficacy, with a higher response rate compared to best supportive care [9]. Alternatives such as oxaliplatin or carboplatin may be considered in patients with concerns about renal function or hearing loss [2, 10]. Gemcitabine monotherapy may be preferred for patients with a poor performance status (PS 2) or other fragility indicators [2]. Despite these treatment options, the prognosis for BTC patients remains poor, with a five-year survival rate of only 4.1% for those with unresectable tumors [11].

In recent years, several RCTs have investigated the combination of first-line chemotherapy with targeted or immunotherapies. The KEYNOTE-966 and TOPAZ-1 trials have recommended the combination of gemcitabine, cisplatin, and immune checkpoint inhibitors (ICI) like Durvalumab or Pembrolizumab as a new first-line treatment approach [12, 13]. Compared to ICI monotherapy, the combination of ICI with other non-ICI therapies may be a better option [14]. Most targeted and immunotherapies are used as second-line treatments for patients with specific pathological characteristics [15–17]. Targeted agents, such as inhibitors of the epithelial growth factor receptor (EGFR) inhibitors and fibroblast growth factor receptor (FGFR) inhibitors, have been tested in combination with standard chemotherapy to improve clinical outcomes for advanced BTC [18, 19].

Although the emergence of new first-line therapies has improved treatment outcomes, the survival benefit remains modest, and disease progression is still observed in the majority of patients [20]. Inappropriate use of these agents not only fails to improve patient survival but may also increase the risk of adverse events [21]. The purpose of this study was to evaluate the efficacy and safety of targeted therapeutics or immunotherapy agents combined with chemotherapy as first-line treatments in patients with advanced BTC. By ranking these therapies based on efficacy and safety, we aim to provide valuable insights to guide clinicians in selecting the most appropriate treatment regimen for individual patients, taking into account their specific pathological characteristics.

## Materials and methods

This NMA was conducted in accordance with the Preferred Reporting Items for Systematic Review and Meta-Analyses (PRISMA) statement [22]. The protocol of this systematic review and NMA was registered on PROSPERO (registration number: CRD42024593872).

### Data sources and search strategy

We searched PubMed, Embase, Cochrane Library and ClinicalTrials.gov using a combination of MeSH terms and free-text keywords. The search focused on clinical trials related to advanced BTC, covering publications from the inception of each database until September 19, 2024 (Supplement S1). Additionally, we reviewed previously published systematic reviews and NMAs to identify any potentially missed studies.

### Selection criteria

The inclusion criteria were defined using the PICOS framework [23].

#### Population

- Histologically or cytologically confirmed diagnosis of locally advanced, recurrent or metastatic biliary tract cancer (intrahepatic or extrahepatic cholangiocarcinoma, gallbladder cancer, or ampullary cancer).
- Patients must not have received previous systemic therapy, though studies where patients received their last adjuvant chemotherapy more than 6 months prior were eligible.

#### Intervention

- First-line treatment with targeted agents or immune checkpoint inhibitors, either alone or in combination with chemotherapy, for advanced biliary tract cancer.

#### Comparator

- Chemotherapy with or without placebo.

#### Outcome

- Primary outcomes: OS and PFS. OS was defined as the time from study day 1 to the date of death due to any cause. PFS was defined as the time from randomization to the first documented disease progression or death, whichever occurred first. Disease progression (PD) was defined as a minimum 20% increase in the sum of the longest diameters (SLD) or the appearance of new lesions.
- Secondary outcomes: ORR and SAE. The ORR was defined as the percentage of patients with confirmed complete response (CR) or confirmed partial response (PR). SAE was defined as an adverse event (AE) resulting in any of the following outcomes: death, a life-threatening condition, persistent or significant disability/incapacity, initial or prolonged inpatient hospitalization, or a congenital anomaly/birth defect.
- Trials were required to report at least one of the primary or secondary outcomes.

#### Study type

- Phase II/III randomized controlled trials (RCTs). If multiple reports from the same trial were available, the most recent results were included.
- No language restrictions were applied.

Studies were excluded if they met at least one of the following exclusion criteria:

- Non-first-line treatments.
- Letters, reviews, case reports, non-human studies, and meta-analyses.
- Trials where patients had received adjuvant chemotherapy within 6 months.
- RCTs with unclear outcome measures.

### Literature selection

All search results were imported into NoteExpress. First, duplicate items were removed. Two researchers independently screened titles and abstracts for eligibility. Trials deemed potentially relevant were reviewed in full text. Disagreements were resolved through discussion, with a third researcher making the final decision if consensus was not reached.

### Data extraction and quality assessment

Two investigators independently extracted data and assessed study quality. Discrepancies were resolved through discussion, and a third investigator was consulted when necessary. The following information was extracted:

1. General characteristics: First author, publication year, trial ID, and study phase.
2. Patient baseline characteristics: Age, region, tumor sites, advanced situation and any specific pathological markers.
3. Treatment details: Intervention arms, sample sizes.
4. Outcome indicators: Primary outcomes (OS and PFS) were extracted as hazard ratios (HRs) and 95% confidence intervals (CIs). Secondary outcomes included ORR and SAEs. For studies reporting Kaplan–Meier curves instead of HRs or CIs, individual patient data (IPD) were extracted using Engauge Digitizer 12.1 [24].

The risk of bias was assessed using the Cochrane Risk of Bias Tool (1.0) for RCTs [25], categorizing studies as “low”, “high”, or “unclear risk”. The Cochrane criteria included random sequence generation, allocation concealment, blinding of participants and personnel, blinding of outcome assessment, incomplete outcome data, selective reporting, and other sources of bias.

### Statistical analysis

The NMA based on Bayesian framework was performed by the “gemtc” package in the R software (v4.3.3). The first and second order fractional polynomial fitting of OS and PFS data was performed by calling JAGS 4.3.1 with R 4.3.3. We evaluated the global heterogeneity by using the I² statistic, where values of < 25%, 25–50%, and >50% indicated low, moderate, and high heterogeneity, respectively [26]. The correlation parameters are estimated by using Markov Chain Monte Carlo (MCMC) model. The study included four chains within a fixed effects framework. The number of iterations was set to 100,000, of which the first 20,000 were used for annealing to eliminate the influence of the initial value. Convergence was assessed using the Gelman-Rubin (R-hat) statistic, where values close to 1 indicated good model convergence. The difference of Deviance Information Criterion (DIC) was performed to evaluate global consistency by comparing their residual deviance between consistency model and inconsistency model [27]. In addition, node splitting was used to assess local inconsistencies when there were closed loops in the network. Evidence within a multi-arm trial is consistent by definition. Thus, if we consider a situation where the evidence structure consists of only multi-arm trials, it cannot be inconsistent [28]. Surfaces under the cumulative ranking (SUCRA) curves were calculated and visualized in ranking plots to show the treatment rankings [29].

Stata (v18.0) software was used to generate evidence relationship diagram and funnel of NMA. In molecular subgroup, the pairwise meta-analysis was conducted using the “meta” package in R software, and risk of bias was assessed using the “robvis” package.

## Results

### Literature search and characteristics of the included studies

**Fig. 1:**
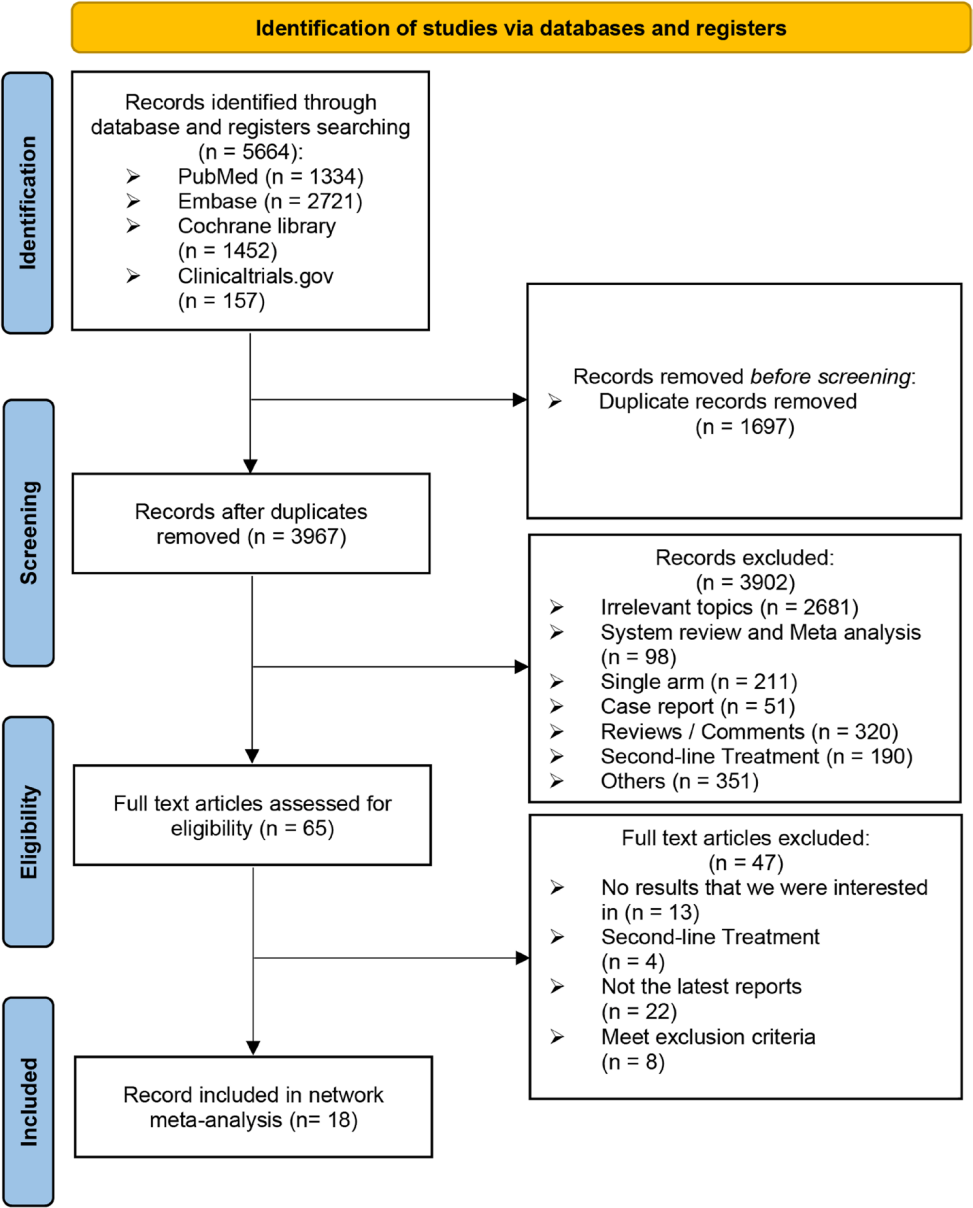
Flowchart of literature search and screening process

We initially identified a total of 5664 records using the search strategy and 1697 duplicates were removed. After screening titles and abstracts, 65 records underwent full-text review. Of these, 22 studies were excluded for presenting outdated findings, 13 for lacking necessary outcomes, 4 for focusing on second-line treatments, 5 for not meeting the control group criteria [30–34], 2 for unrelated regimens [35, 36], and 1 for including patients with prior systemic therapy [37]. Finally, 18 studies met the inclusion criteria (Fig. 1). The drugs and their targets are detailed in Supplement S2. To minimize heterogeneity, studies were divided into two groups based on pathological characteristics: the “molecular subgroup” and the “average group”. Three studies overlapped, providing data for each.

In average group, the 15 RCTs included a total cohort of 3692 patients with advanced BTC. The chemotherapy (Chemo) regimens included gemcitabine plus cisplatin (GC) in nine cases, gemcitabine plus oxaliplatin (GO) in four cases, and gemcitabine alone (G) in two cases. The control group included six studies with a placebo. Tumor sites are summarized in Supplement S3. Among the included studies, only one exclusively enrolled patients with metastatic BTC, while the others included patients with locally advanced or recurrent diseases. Overall, the demographic characteristics of the included trials showed a high degree of comparability. Basic features of included studies were summarized in Table. Network evidence diagrams illustrating the relationships between the different interventions were provided in Supplement S4. We calculated the percentage of each SAE and focused on those with a total percentage exceeding 5% across groups. A heat map illustrating the frequency of various SAEs was provided in Supplement S5.

In molecular subgroup, we extracted data on nine different characteristics from six RCTs, as one of these studies provided a detailed subgroup analysis fully reporting the results for the various pathological characteristics we required. Owing to missing data and patient overlap, we conducted a pairwise meta-analysis exclusively. The chemotherapy regimens included GC and GO. Basic features of included studies were summarized in Table.

### Risk of bias assessment

Overall, the included 18 RCTs were of high quality, despite comprising four meeting abstracts [18, 38–40] and one preprint version [41]. The primary source of high-risk bias was in the domain of blinding of participants and personnel, due to the open-label design. Two studies were prematurely terminated. The sponsor decided to discontinue the first study due to the unlikelihood of achieving the primary objective of overall survival [42]. The second study was discontinued following the sponsor’s decision to halt the development of infigratinib for oncology indications within their territory [18]. The summary of bias was summarized and the detailed assessment of each study can be found in Supplement S6.

### Heterogeneity, consistency and convergence

In average group, the heterogeneity was low across the studies for both primary and secondary outcomes when using a fixed-effects model (I^2^ < 25%) and the differences in DIC values were minimal, indicating a high level of global consistency, as shown in Supplement S7. The R-hat value was close to 1, suggesting good model convergence. There was no publication bias in the included studies, as indicated by the symmetrical distribution of effect sizes in the funnel plots, presented in the Supplement S8. In molecular subgroup, the I² statistic was high in the KRAS wild-type subgroup for the outcomes of PFS and OS. A leave-one-out sensitivity analysis was performed in PFS, revealing that the heterogeneity primarily originated from A. Vogel 2018 [43].

### Network meta-analysis of average group

**Fig. 2:**
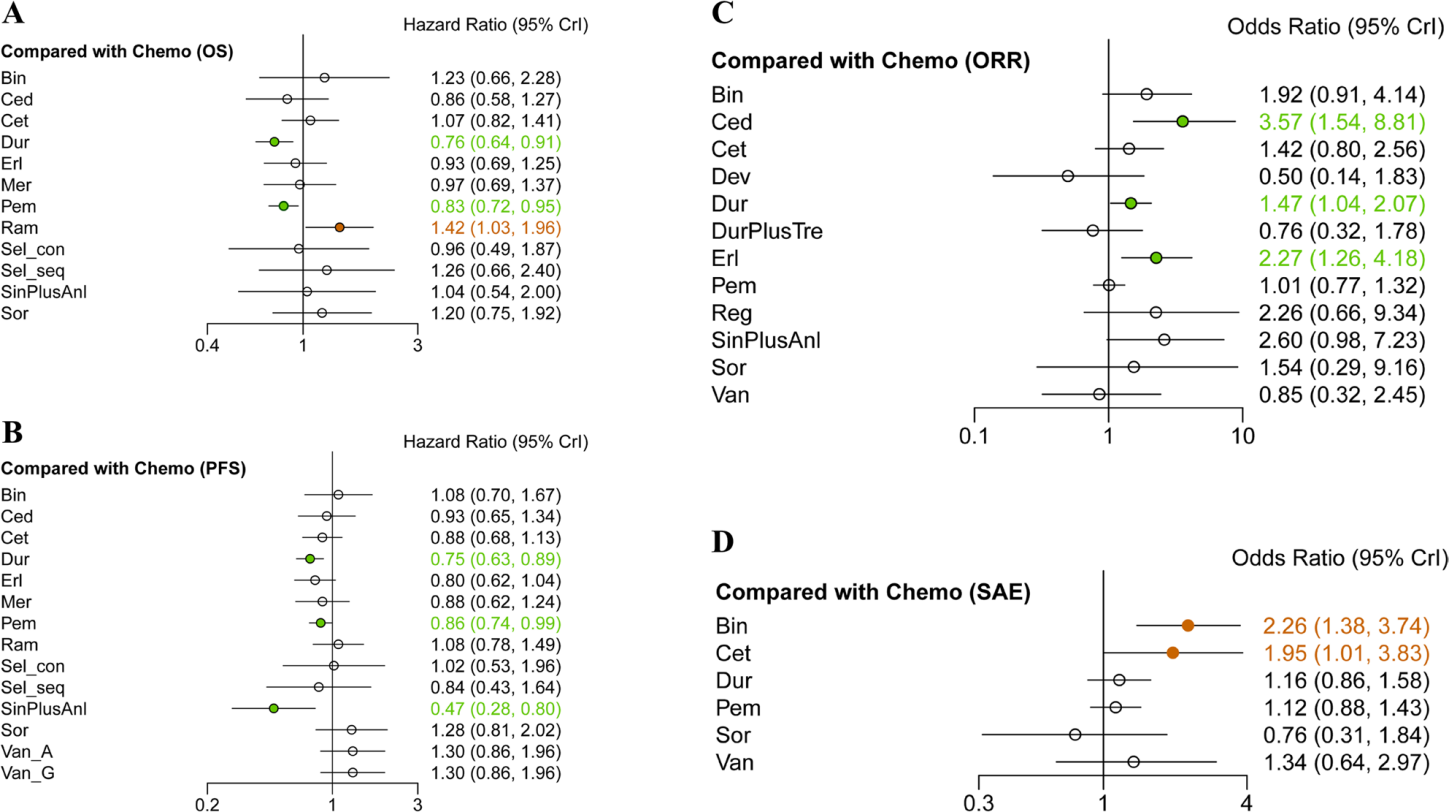
Forest plots of outcomes compared with chemotherapy in average group. **A** Forest plots of OS compared with chemotherapy; (**B**) Forest plots of PFS compared with chemotherapy; (**C**) Forest plots of ORR compared with chemotherapy; (**D**) Forest plots of SAE compared with chemotherapy. All regimens include chemotherapy, except for Van_A. Abbreviations: Sin: Sintilimab; Anl: Anlotinib; Dev: Devimistat; Bin: Bintrafusp alfa; Pem: Pembrolizumab; Dur: Durvalumab; Tre: Tremelimumab; Ram: Ramucirumab; Mer: Merestinib; Reg: Regorafenib; Cet: Cetuximab; Erl: Erlotinib; Ced: Cediranib; Sor: Sorafenib; Sel_con: continuous Selumetinib; Sel_seq: sequential Selumetinib; Van_A: Vandetanib alone; Van_G: Vandetanib combined with gemcitabine; Van: Van_A and Van_G

**Fig. 3:**
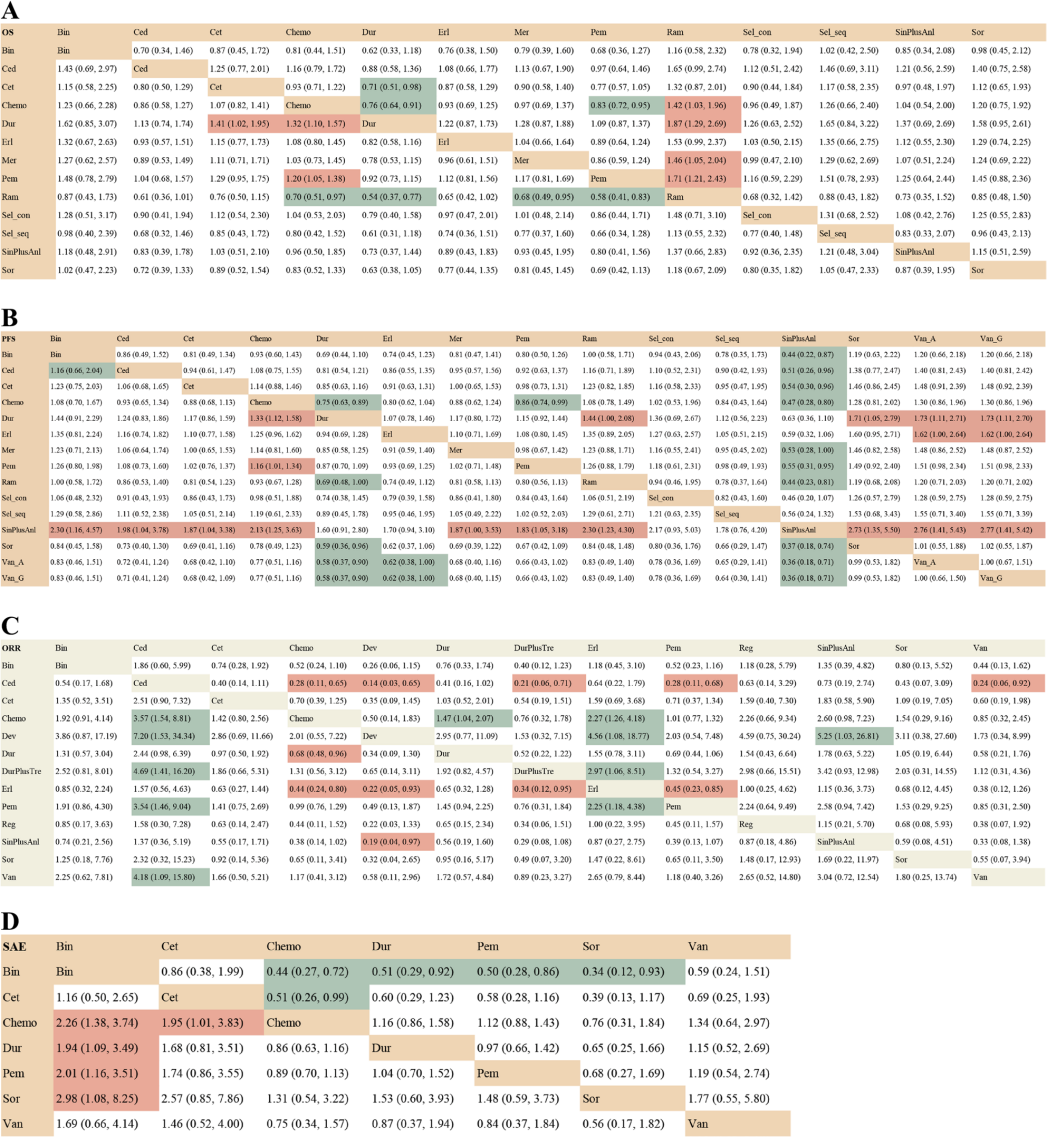
Network league table. **A** Hazard Ratios (HR) and 95% confidence interval (CIs) for OS; (**B**) HR and 95% CIs for PFS; (**C**) odds ratios (OR) and 95% CIs for ORR; (**D**) OR and 95% CIs for SAE

**Fig. 4:**
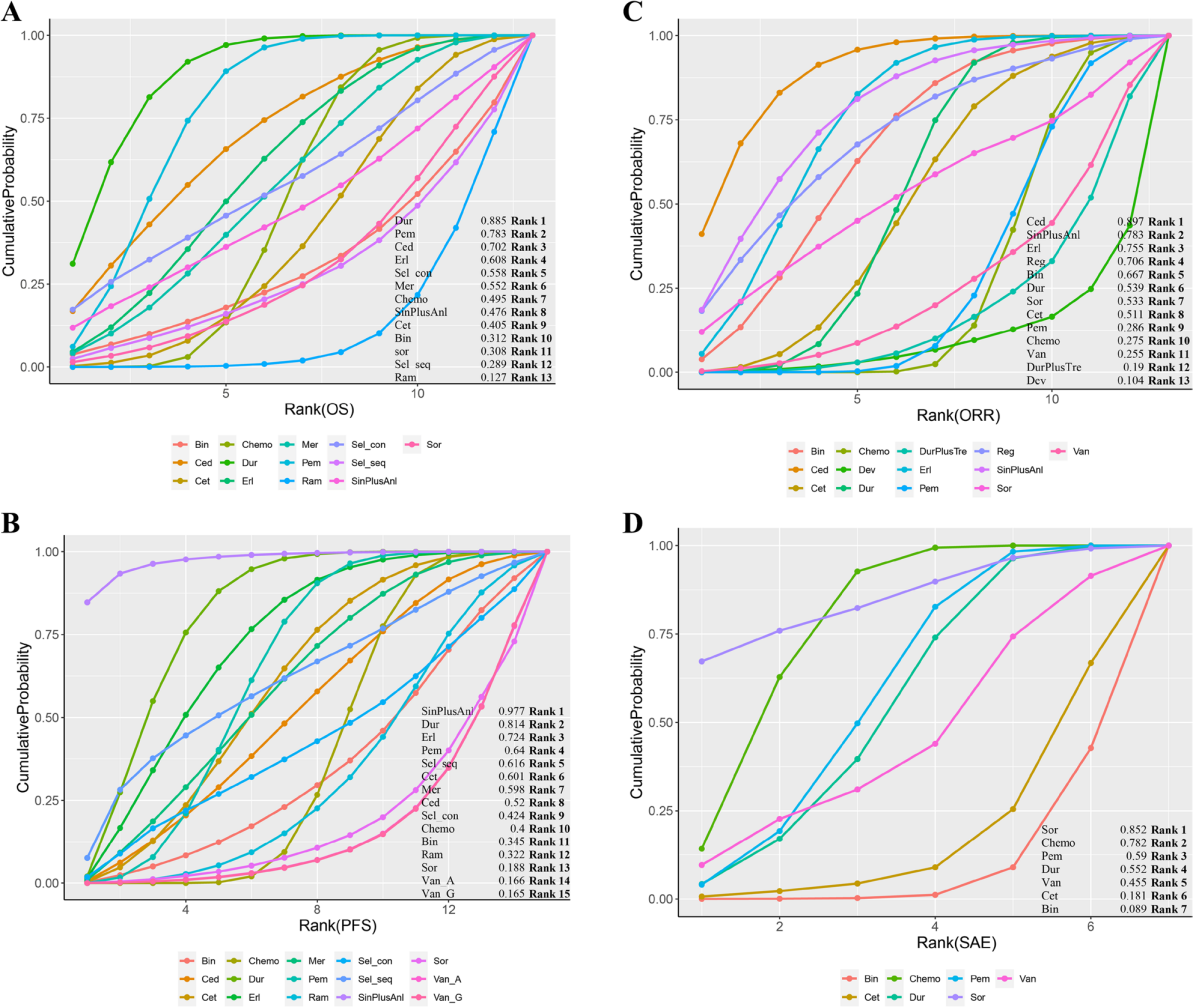
The surface under the cumulative ranking (SUCRA) diagrams. **A** SUCRA diagrams of OS; (**B**) SUCRA diagrams of PFS; (**C**) SUCRA diagrams of ORR; (**D**) SUCRA diagrams of SAE

Regarding OS, 11 trials reported the primary outcomes of OS. Pembrolizumab and Durvalumab had a statistically significant difference in prolonging the OS compared to chemotherapy (HR 0.83, 95% CI 0.72–0.95; HR 0.76, 95% CI 0.64–0.91), while others were comparable to chemotherapy except Ramucirumab (HR 1.42, 95% CI 1.03–1.96) (Fig. 2A). Meanwhile, Durvalumab had a statistically significant difference in prolonging the OS compared to Cetuximab (HR 0.71, 95% CI 0.51–0.98) and Pembrolizumab showed a trend toward prolonging the OS compared to Cetuximab (HR 0.77, 95% CI 0.57–1.05). Unfortunately, Ramucirumab was associated with a reduced OS compared to other treatments (Fig. 3A). According to Bayesian ranking profiles, the SUCRA values of all 11 treatment regimens was shown (Fig. 4A). Durvalumab ranked highest (0.885), followed by Pembrolizumab (0.783) and Cediranib (0.702). Ramucirumab ranked last (0.127).

In terms of PFS, 12 trials reported the primary outcomes of PFS. Notably, patients treated with Sintilimab plus Anlotinib were more likely to experience an improvement than those who received others. As with OS, Pembrolizumab and Durvalumab significantly prolonged PFS compared to chemotherapy (HR 0.86, 95% CI 0.74–0.99; HR 0.75, 95% CI 0.63–0.89), while Erlotinib demonstrated a modest advantage in PFS (HR 0.80, 95% CI 0.62–1.04) (Fig. 2B). Durvalumab showed a significant difference in prolonging PFS compared to ramucirumab, sorafenib and vandetanib. Erlotinib also showed a significant advantage over Vandetanib (Fig. 3B). The top three regimens based on SUCRA values were Sintilimab plus Anlotinib, Durvalumab, and Erlotinib (0.977, 0.814, and 0.724, respectively), with Vandetanib ranked lowest (0.165) (Fig. 4B).

Regarding ORR, 13 trials reported the secondary outcomes of ORR. Cediranib, Durvalumab and Erlotinib were observed to have significantly better efficacy compared to chemotherapy (HR 3.57, 95% CI 1.54–8.81; HR 1.47, 95% CI 1.04–2.07; HR 2.27, 95% CI 1.26–4.18) (Fig. 2C). The results of both direct and indirect comparisons are summarized in a league table (Fig. 3C). Ultimately, considering the comparative effects of all treatment regimens on ORR, Cediranib, Sintilimab plus Anlotinib, and Erlotinib ranked as the top three treatments based on the SUCRA values (0.897, 0.783, and 0.755, respectively), while Devimistat ranked lowest (0.104) (Fig. 4C).

In terms of SAE, 6 trials reported the secondary outcomes of SAE. No regimens demonstrated a significant safety advantage over chemotherapy, while bintrafusp alfa and cetuximab showed no safety benefits compared to chemotherapy (HR 2.26, 95% CI 1.38–3.74; HR 1.95, 95% CI 1.01–3.83) (Fig. 2D). Durvalumab, pembrolizumab and Sorafenib had a more positive effect than Bintrafusp alfa in SAE (HR 0.51, 95% CI 0.29–0.92; HR 0.50, 95% CI 0.28–0.86; HR 0.34, 95% CI 0.12–0.93) (Fig. 3D). The safest regimens were observed to be Sorafenib, Chemotherapy and Pembrolizumab (0.852, 0.782 and 0.590, respectively), while Bintrafusp alfa was ranked at the bottom (0.089) (Fig. 4D).

### Pairwise meta-analysis of molecular subgroup

**Fig. 5:**
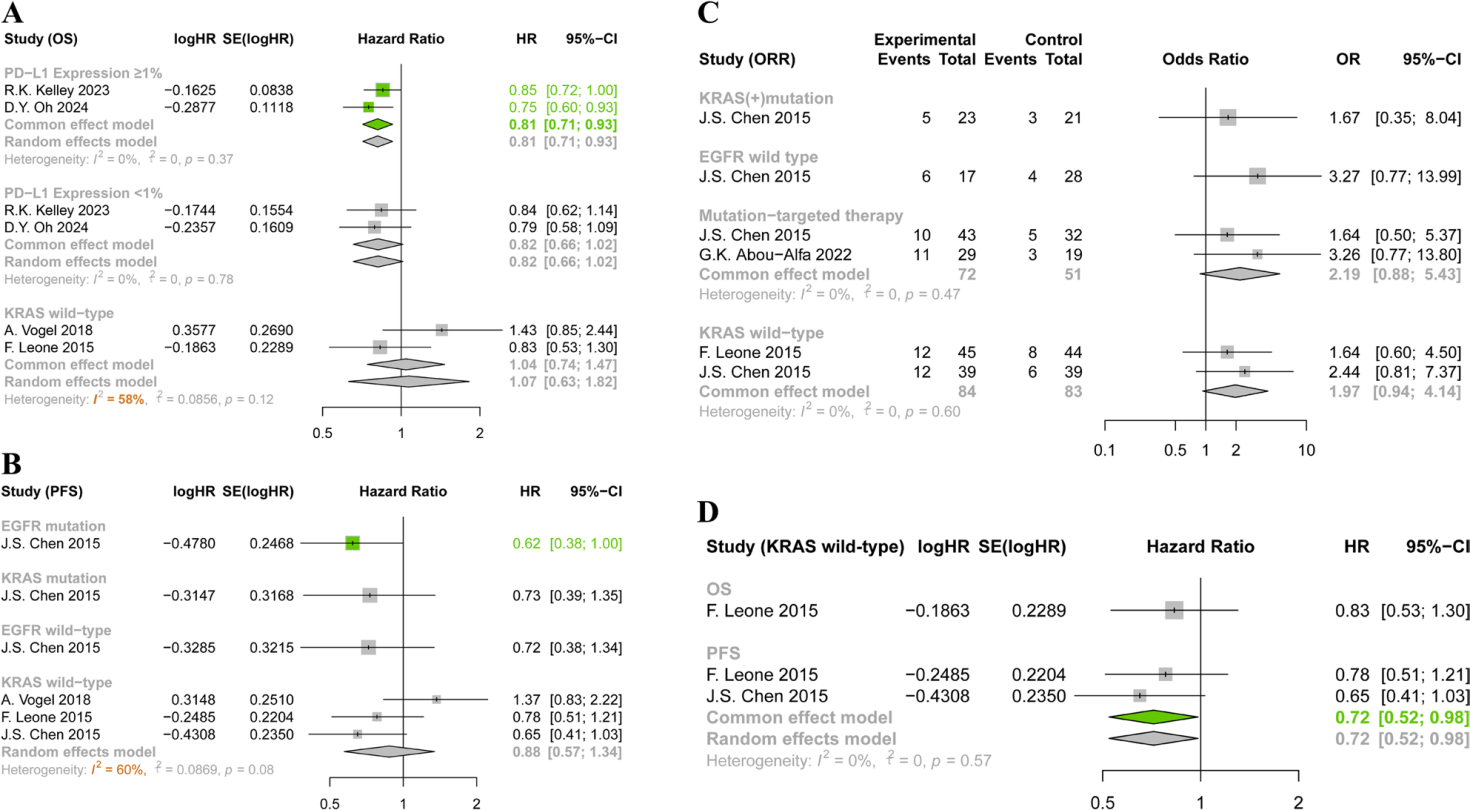
Forest plots in molecular subgroup. **A** Forest plots of OS; (**B**) Forest plots of PFS; (**C**) Forest plots of ORR; (**D**) Forest plots of OS and PFS for patients with KRAS wild-type

We extracted data on nine distinct characteristics from six RCTs. Based on these characteristics, we further categorized the data into various subgroups and conducted pairwise meta-analyses for each subgroup. The results are presented in detail through forest plots (Fig. 5). The results showed that patients with PD − L1 Expression ≥ 1% benefited from ICI regimens (Pembrolizumab or Durvalumab) in terms of OS, as assessed using a fixed-effects model (HR 0.81, 95% CI 0.71–0.93). Among the three studies that included patients with KRAS wild-type, no significant benefits were observed in OS, PFS, or ORR. After excluding A. Vogel 2018, the results indicated that the use of EGFR inhibitors (Cetuximab and Panitumumab) significantly prolonged PFS for patients with KRAS wild-type, with a trend toward higher OS and ORR as well. Furthermore, two targeted drugs (Cetuximab and Infigratinib) showed nearly statistically significant improvements in terms of ORR (OR 2.19, 95% CI 0.88–5.43) in mutation-targeted therapy subgroup. Similarly, in patients with EGFR mutations treated with Cetuximab, PFS was significantly prolonged (HR 0.62, 95% CI 0.38–1.00.38.00).

## Discussion

To the best of our knowledge, this is the first Bayesian network meta-analysis comparing the efficacy and safety of targeted therapeutics or immunotherapy agents combined with chemotherapy as first-line treatments for advanced BTC. Notably, our study provides a more comprehensive assessment compared to recent similar systematic reviews by enabling indirect comparisons and treatment ranking among various combination regimens, analyzing all four key outcomes—OS, PFS, ORR, and SAEs—and conducting subgroup analyses based on KRAS status and PD-L1 expression, thereby offering unique insights into biomarker-driven treatment responses and advancing the field towards precision oncology [44–46].

We conducted a rigorous quality assessment and provided a detailed description of the inclusion criteria and outcome measures. The results indicated that Pembrolizumab or Durvalumab combined with chemotherapy significantly prolonged OS and PFS compared to chemotherapy alone. Moreover, Sintilimab plus Anlotinib combined with chemotherapy demonstrated superior efficacy in prolonging PFS compared to Pembrolizumab or Durvalumab combined with chemotherapy. Importantly, there was no significant difference in SAE between Pembrolizumab or Durvalumab combined with chemotherapy and chemotherapy alone, indicating that such combinations are promising as first-line treatments for advanced BTC. However, since most clinical studies focusing on specific pathological characteristics primarily investigate second-line treatments, our data for molecular subgroup were incomplete. As a result, we conducted only a pairwise meta-analysis in molecular subgroup.

The latest NCCN and ESMO clinical practice guideline recommend Durvalumab or Pembrolizumab combined with GC as the preferred first-line treatment for advanced BTC [47, 48]. Notably, Pembrolizumab combined with GC has been newly included in these guidelines [2, 49]. The KEYNOTE-966 and TOPAZ-1 trials share many similarities and are the only two global studies demonstrating a significant prolongation of OS with PD-1 inhibitors in patients with advanced BTC [12, 13]. However, according to the subgroup analyses from the TOPAZ-1 and KEYNOTE-966 trials, neither durvalumab nor pembrolizumab showed a significant survival benefit in the PD-L1 < 1% subgroup. Notably, patients with PD-L1 ≥ 1% appeared to derive the greatest benefit from immunotherapy, suggesting that PD-L1 expression may serve as a potential biomarker for predicting treatment response. Moreover, more than 57% of patients in the TOPAZ-1 trial and over 68% in the KEYNOTE-966 trial had PD-L1 expression ≥ 1%, further supporting the potential utility of PD-L1 as a predictive biomarker in guiding immunotherapy selection.

Inspiringly, our analysis also revealed that Sintilimab plus Anlotinib combined with GC outperformed Pembrolizumab or Durvalumab combined with GC in terms of PFS. Sintilimab is a PD-1 inhibitor, while Anlotinib is an oral multi-target tyrosine kinase inhibitor (TKI) that targets VEGFR1-3, PDGFRα/β, FGFR1-4, c-Kit, RET, and c-FMS. By inhibiting these key receptors, Anlotinib blocks downstream signaling pathways, effectively suppressing tumor proliferation, angiogenesis, migration, and metastasis [50]. Given that VEGF and its receptors are overexpressed in 40–75% of BTC patients, and this overexpression correlates with metastasis and poor prognosis, Anlotinib’s potential therapeutic benefit in BTC is significant [51, 52]. However, regarding OS, Sintilimab plus Anlotinib combined with GC showed no significant advantage over GC alone. Potential explanations include a higher proportion of patients in the chemotherapy group receiving subsequent anti-tumor therapies, or that the initial dosage of Anlotinib may have been too high. The results of our NMA are consistent with IMbrave-151 trial, which indicated that for PFS, the combination of Atezolizumab (PD-1 inhibitor) and Bevacizumab (anti-VEGF antibody) with chemotherapy showed a trend toward being superior to Atezolizumab with chemotherapy (HR 0.76; 95% CI: 0.51–1.14) [31]. Although this single-center randomized controlled trial included only 80 patients, the findings still provide valuable evidence for the efficacy and feasibility of combining PD-1 antibody, vascular-targeted TKI, and GC as a first-line treatment for advanced BTC. Nevertheless, while Cediranib (a vascular-targeted TKI) combined with chemotherapy demonstrated superior ORR compared to chemotherapy alone, the combination of Cediranib with chemotherapy did not show significant statistical differences in survival outcomes [53]. Furthermore, the OS of Ramucirumab (anti-VEGFR-2 antibody) combined with chemotherapy was even lower than that of chemotherapy alone [54]. Larger-scale global trials are needed to validate these findings.

EGFR expression has been reported in 67–100% of biliary tract cancers [43]. KRAS is a downstream molecule in the EGFR pathway. Panitumumab has been shown to confer clinical benefit in patients with metastatic colorectal cancer and wild-type KRAS [55]. According to a meta-analysis, EGFR receptor antagonists did not demonstrate a sustained survival benefit compared to chemotherapy alone [43]. However, two studies reported that the combination of Erlotinib or Cetuximab with chemotherapy demonstrated an improved ORR compared to chemotherapy alone, while PFS showed a trend towards potential benefit, approaching statistical significance, compared to chemotherapy alone [19, 56]. For KRAS wild-type patients, Cetuximab or Panitumumab combined with chemotherapy showed a trend towards improved OS and ORR compared to chemotherapy alone, while PFS demonstrated a statistically significant benefit. The latest Chinese guidelines also recommend gemcitabine combined with oxaliplatin and Erlotinib (strong recommendation) or gemcitabine combined with oxaliplatin and Panitumumab (weak recommendation) for patients with an acceptable performance status of 1–2 (PS 1–2), particularly for those with KRAS wild-type [57]. FGFR2 fusions are identified as driver events in 10–15% of intrahepatic cholangiocarcinoma [58]. Single-arm study have already demonstrated the clinical benefits of FGFR inhibitor (Futibatinib) in previously treated patients with FGFR2 fusion or rearrangement-positive intrahepatic cholangiocarcinoma [59]. However, in first-line treatment, the trial showed that monotherapy with FGFR inhibitor (Infigratinib) did not significantly outperform chemotherapy in terms of ORR [18]. The combination of FGFR inhibitors and chemotherapy remains promising in the first-line treatment of advanced BTC patients with FGFR mutations. Additionally, two clinical trials comparing FGFR inhibitors with chemotherapy as first-line treatments (ClinicalTrials.gov IDs: NCT03656536 and NCT04093362) have yet to report their final results.

Currently, the optimal combination strategies between various targeted agents, immune checkpoint inhibitors, and chemotherapy for treating advanced BTC remain unclear. Based on existing evidence, this study indirectly compared multiple first-line treatment regimens in terms of their efficacy and safety, providing a reference for clinical decision-making. Furthermore, Subgroup analyses were carried out stratified by molecular characteristics, preliminarily revealing the potential predictive value of biomarkers such as KRAS wild-type status and PD-L1 in treatment response. This provides a theoretical basis for the development of precision oncology. However, most current phase III trials evaluating first-line therapies have not been stratified based on molecular characteristics, resulting in a lack of high-quality evidence tailored to specific patient populations. Therefore, there is an urgent need to develop robust biomarkers that can accurately predict which patients are most likely to benefit from treatment and to promote more prospective clinical trials guided by such biomarkers.

Looking ahead, with advancements in molecular profiling technologies and novel drug development, the first-line treatment landscape for BTC is expected to evolve into a truly “molecularly driven” era. Targeted therapies against genetic alterations such as KRAS and FGFR mutations will be further validated in larger-scale clinical trials. At the same time, combination regimens involving immunotherapy, targeted therapy, and chemotherapy are likely to become major research focuses (Table 1).

**Table 1:** Basic features of included studies in average group

Study	Journal of publication	Age（median）	Region	Tumor sites	Advancedsituation	size	Treatment arms	OS-HR (95% CI)	PFS-HR (95% CI)	ORR	SAE
J. Li 2024 [41]NCT04300959RCT II^a^	Nature Portfolio	A:62.5B:59.5	China	IHC 50 EHC 11GBC 19	Locally AdvancedMetastasesRecurrent	80	A: sintilimab plus anlotinib + GC (40) B: GC (40)	1.04(0.40–1.49.40.49)	0.47(0.22–0.64.22.64)	A:19/37B:10/34	NA
V. Sahai 2024 [38]NCT04203160RCT Ⅰ/Ⅱ^b^	Journal of Clinical Oncology	NA	USA	IHC 48EHC 13GBC 7	Locally AdvancedMetastatic	75	A：Devimistat + GC (57)B：GC (18)	NA	NA	A:10/34B:6/13	NA
D.Y. Oh 2024 [42]NCT04066491RCT II/Ⅲ	Hepatology	A：64B：65	Multiple countries	IHC 132EHC 66GBC 78APC 21	Locally AdvancedMetastatic	297	A: Bintrafusp alfa + GC (148)B：Placebo + GC (149)	1.23(0.66–2.28.66.28)	1.08(0.70–1.66)	A:23/73B:15/77	A:58/146 B:36/149
R.K. Kelley 2023 [12]NCT04003636RCT Ⅲ	The Lancet Oncology	A:64B:63	Multiple countries	IHC 633EHC 203GBC 233	Locally AdvancedMetastatic	1069	A: Pembrolizumab + GC (533)B: Placebo + GC (536)	0.83(0.72–0.95.72.95)	0.86(0.75–1.00.75.00)	A:153/533B:153/536	A:276/529B:263/534
D.Y. Oh 2024 [13]NCT03875235RCT Ⅲ	The Lancet Gastroenterology and Hepatology	A:64B:64	Multiple countries	IHC 383EHC 131GBC 171	Locally AdvancedMetastaticRecurrent	685	A：Durvalumab + GC（341）B：Placebo + GC (344)	0·76(0.64–0.91.64.91)	0.75(0.63–0.89.63.89)	A:91/341B:64/343	A:160/338B:149/342
A. Vogel 2022 [39]NCT03473574RCT II^b^	Annals of Oncology	NA	Germany	IHC 98EHC 27 GBC 13	Metastatic	138	A: Durvalumab plus Tremelimumab + G/GC (22/52)B: GC (35)C: Durvalumab + GC (29)	NA	NA	A:13/74B:10/35C:6/29	NA
J.W. Valle 2021 [54]NCT02711553RCT II	The Lancet Oncology	NA	Multiple countries	IHCEHCGBC	Locally AdvancedMetastaticRecurrent	294	A: Ramucirumab + GC (103)B: Merestinib + GC (95)C: Pooled Placebo + GC (96)	1.42 (1.03–1.96.03.96)0.97(0.69–1.37.69.37)	1.08(0.78–1.49.78.49)0.88(0.63–1.25.63.25)	NA	NA
E. Assenat 2021 [40]NCT02386397RCT II^b^	Annals of Oncology	NA	France	IHC 39EHC 13 GBC 11	Locally AdvancedMetastaticRecurrent	63	A：Regorafenib + GO (42)B：GO (21)	NA	NA	A:14/42B:4/21	NA
Mark K. Doherty 2022 [60]NCT02151084RCT II	British Journal of Cancer	A:60B:61C:64	Canada	IHC 22EHC 16GBC 19	Locally AdvancedMetastatic	57	A: continuous selumetinib + GC (n=19)B: sequential selumetinib + GC (n=19)C: GC (n=19)	0.96(0.49–1.88.49.88)1.26(0.66–2.40.66.40)^c^	1.02(0.53–1.95.53.95)0.84(0.43–1.64.43.64)^c^	NA	NA
J.S. Chen 2015 [19]NCT01267344RCT II	Annals of Oncology	A:61B:59	China	IHC 89EHC 19GBC 14	Locally AdvancedMetastatic	122	A: cetuximab + GO (62)B: GO(60)	0.99(0.66–1.50.66.50)^c^	0.70(0.48–1.01.48.01)	A:17/62B:9/60	NA
J. Lee 2012 [56]NCT01149122RCT Ⅲ	The Lancet Oncology	A:59B:61	Korea	IHC/EHC 180GBC 82 APC 6	MetastaticRecurrent	268	A：erlotinib + GO (n=135)B：GO (n=133)	0.93(0.69–1.25.69.25)	0·80(0.61–1.03.61.03)	A:40/135B:21/133	NA
J.W. Valle 2015 [53]NCT00939848RCT II	The Lancet Oncology	A:68B:64.5	United Kingdom	IHC 29EHC 48 GBC 39APC 8	Locally AdvancedMetastaticRecurrent	124	A：cediranib + GC (62)B: Placebo+GC (62)	0.86(0.58–1.27.58.27)	0.93(0.65–1.35.65.35)	A:26/59B:10/54	NA
A. Santoro 2015 [61]NCT00753675RCT II	Annals of Oncology	NA	Italy	IHC 87EHC 39 GBC 31 unspecified 1Periamp 15	Locally AdvancedMetastatic	173	A: Vandetanib (59)B: Vandetanib+ G (58)C: Placebo+G (56)	NA	1.30(0.86–1.96.86.96)1.30(0.75–1.70.75.70)	A:2/56B:11/57C:7/52	A:16/59B:15/58C:12/56
D. Malka 2014 [62]NCT00552149RCT II	The Lancet Oncology	A:61B:62	France	IHC 95EHC 22GBC 22 APC 1Multifocal 2unspecified 8	Locally AdvancedMetastatic	150	A: cetuximab + GO (n=76)B: GO (n=74)	1.14(0.80–1.63.80.63)^c^	1.07(0.76–1.50.76.50)^c^	A:18/76B:17/74	A:39/76B:25/71
M. Moehler 2014 [63]NCT00661830RCT II	European Journal of Cancer	A：64B：64.5	Germany	IHC 62 EHC 22GBC 13	Locally AdvancedMetastatic	97	A: Sorafenib + G (49)B: Placebo + G (48)	1.20(0.75–1.93.75.93)	1.28(0.81–2.02.81.02)	A:4/28B:3/30	A：33/49B：35/48

*Abbreviations*: *IHC* Intrahepatic cholangiocarcinoma, *EHC* Extrahepatic cholangiocarcinoma, *GBC* Gallbladder cancer, *APC* Ampullary cancer

^a^Preprint version

^b^Meeting abstract

^c^Date extracted from Kaplan–Meier curves

This analysis has several limitations. Owing to the limited number of RCTs comparing targeted therapeutics or immunotherapy agents with chemotherapy as first-line treatments for advanced BTC, an effective closed-loop network could not be established. Additionally, some studies have not provided details on the specific pathological characteristics. We only performed a pairwise meta-analysis in molecular subgroup. We used a fixed-effect model, and while heterogeneity was minimal, this may still have led to an overly optimistic effect estimate. Furthermore, the ROB-1 tool was used to assess the quality of the included studies. While we incorporated unpublished clinical trial and extracted some data from Kaplan-Meier curves, neither ROB-1 nor ROB-2 provides relevant criteria for evaluating potential biases related to these aspects. A more comprehensive evaluation tool will be required for a thorough quality assessment of the literature. BTC comprises multiple anatomical subtypes, which exhibit significant biological heterogeneity. This study analyzed these subtypes collectively, which may have affected the accurate identification of survival benefits associated with specific treatment regimens. Future research should focus on more precise patient selection in prospective clinical trials to overcome this limitation, thereby generating more targeted and clinically meaningful results (Table 2).

**Table 2:** Basic features of included studies in molecular subgroup

Study	Journal of publication	Age（median）	Region	Tumor sites	Advancedsituation	size	Treatment arms	OS-HR (95%CI)	PFS-HR (95% CI)	ORR	SAE	Note
R.K. Kelley 2023 [12]NCT04003636RCT Ⅲ	The Lancet Oncology	NA	Multiple countries	NA	NA	728	A: Pembrolizumab + GC (363)B: Placebo + GC (365)	0.85(0.72–1.00.72.00)	NA	NA	NA	PD-L1 expression ≥1%
R.K. Kelley 2023 [12]NCT04003636RCT Ⅲ	The Lancet Oncology	NA	Multiple countries	NA	NA	223	A: Pembrolizumab + GC (113)B: Placebo + GC (110)	0.84 (0.62–1.14)	NA	NA	NA	PD-L1 expression <1%
D.Y. Oh 2024 [13]NCT03875235RCT Ⅲ	The Lancet Gastroenterologyand Hepatology	NA	Multiple countries	NA	NA	406	A: Durvalumab + GC (199)B: Placebo + GC (207)	0.75(0.60–0.93.60.93)	NA	NA	NA	PD-L1 expression ≥1%
D.Y. Oh 2024 [13]NCT03875235RCT Ⅲ	The Lancet Gastroenterologyand Hepatology	NA	Multiple countries	NA	NA	206	A: Durvalumab + GC (103)B: Placebo + GC (103)	0.79(0.58–1.09.58.09)	NA	NA	NA	PD-L1 expression <1%
A. Vogel 2018 [43]NCT01320254RCT II	European Journal of Cancer	A:62B:59.5	Germany	IHC 61EHC 24GBC 14	Locally AdvancedMetastatic	90	A: Panitumumab + GC (62)B: GC (28)	1.43(0.85–2.44.85.44)	1.37(0.83–2.22.83.22)	NA	NA	KRAS wild-type
F. Leone 2015 [64]NCT01389414RCT II	Cancer	NA	Italy	IHC 42EHC 19GBC 28	Locally AdvancedMetastatic	89	A: Panitumumab + GO (45)B: GO (44)	0.83(0.53–1.3.53.3)	0.78(0.51–1.21.51.21)	A:12/45B: 8/44	A: 18/45B: 12/44	KRAS wild-type
J.S. Chen 2015 [19]NCT01267344RCT II	Annals of Oncology	NA	China	NA	NA	78	A: Cetuximab + GO (39)B: GO (39)	NA	0.65(0.41–1.03.41.03)	A:12/39B: 6/39	NA	KRAS wild-type
J.S. Chen 2015 [19]NCT01267345RCT II	Annals of Oncology	NA	China	NA	NA	45	A: Cetuximab + GO (17)B: GO (28)	NA	0.72(0.38–1.34.38.34)	A:6/17B:4/28	NA	EGFR wild-type
J.S. Chen 2015 [19]NCT01267344RCT II	Annals of Oncology	NA	China	NA	NA	44	A: Cetuximab + GO (23)B: GO (21)	NA	0.73(0.39–1.35.39.35)	A:5/23B:3/21	NA	KRAS mutation
G.K. Abou-Alfa 2022NCT03773302 [18]RCT Ⅲ^b^	Journal of Clinical Oncology	NA	Multiple countries	IHC 45EHC 3	Locally AdvancedMetastatic	48	A：Infigratinib （29）B：GC (19)	NA	NA	A:11/29B：3/19	A:10/29 B：0/17	FGFR mutation
J.S. Chen 2015 [19]NCT01267344RCT II	Annals of Oncology	NA	China	NA	NA	75	A: Cetuximab + GO (43)B: GO (32)	NA	0.62(0.38–1.00.38.00)	A:10/43B: 5/32	NA	EGFR mutation

*Abbreviations*: *IHC* Intrahepatic cholangiocarcinoma, *EHC* Extrahepatic cholangiocarcinoma, *GBC* Gallbladder cancer, *APC* Ampullary cancer

^a^Preprint version

^b^Meeting abstract

^c^Date extracted from Kaplan–Meier curves

## Supplementary Information

Supplementary Material 1. 

## Data Availability

Data is provided within the manuscript or supplementary information files.
