# Supplementary material for: Comparative efficacy and safety of targeted therapeutics or immunotherapy agents combined with chemotherapy as first-line treatment for advanced biliary tract cancer: a systematic review and network meta-analysis
Source: BMC Cancer. 2025 Dec 3;26:51. doi: 10.1186/s12885-025-15370-8 (PMC12798126; doi:10.1186/s12885-025-15370-8)
Supplement: Supplementary file 1 — Supplementary Material 1. [file 12885_2025_15370_MOESM1_ESM.docx]

**Supplementary Materials**

Supplement S1: Search strategies

Supplement S2: The drugs and their targets mentioned in the studies

Supplement S3: Tumor sites in average group

Supplement S4: Network evidence diagrams

Supplement S5: Heat map of SAE

Supplement S6: Risk of bias assessment

Supplement S7: I^2^ and DIC differences

Supplement S8: Funnel plots

Supplement S9: Basic features of included studies

**Supplement S1: Search strategies**

**(A) Pubmed**

| No. | Query | Results |
| --- | --- | --- |
| #1 | "Cholangiocarcinoma"[Mesh] OR "Bile Duct Neoplasms"[Mesh] OR "Gallbladder Neoplasms"[Mesh] OR "Biliary Tract Neoplasms"[Mesh] | 37,085 |
| #2 | cholangio* OR bile duct OR biliary OR gallbladder OR ampullary | 184,392 |
| #3 | carcinoma* OR cancer* OR tumor* OR neoplasm* OR malign* | 4,107,509 |
| #4 | #2 AND #3 | 55,804 |
| #5 | #1 OR #4 | 67,344 |
| #6 | "Immune Checkpoint Inhibitors"[Mesh] OR "Drug Therapy"[Mesh] OR "Immunotherapy"[Mesh] OR "Molecular Targeted Therapy"[Mesh] | 1,850,301 |
| #7 | Checkpoint Inhibitors, Immune OR Immune Checkpoint Blockers OR Checkpoint Blockers, Immune OR Immune Checkpoint Inhibitor OR  Checkpoint Inhibitor, Immune OR Immune Checkpoint Blockade OR Checkpoint Blockade, Immune OR Immune Checkpoint Inhibition OR Checkpoint Inhibition, Immune | 19,540 |
| #8 | Chemotherapy OR chemo-therapy OR Chemotherapies OR Pharmacotherapy OR Pharmacotherapies OR Therapy, Drug OR Drug Therapies  OR Therapies, Drug | 531,179 |
| #9 | Immunotherapies OR Molecular Targeted Therapies OR Targeted Therapy, Molecular OR Therapy, Molecular Targeted OR  Targeted Molecular Therapy OR Molecular Therapy, Targeted OR Targeted Molecular Therapies OR Therapy, Targeted Molecular | 19,803 |
| #10 | #6 OR #7 OR #8 OR #9 | 2,220,324 |
| #11 | Randomized Controlled Trial OR controlled clinical trial OR randomized OR randomised OR randomly OR trial OR phase | 2,671,639 |
| #12 | first line OR first-line OR untreated OR treatmentnaive OR treatment-naïve OR treatment OR therapy | 6,986,460 |
| #13 | #5 AND #10 AND #11 AND #12 | 1,334 |

**(B) Embase**

| No. | Query | Results |
| --- | --- | --- |
| #1 | 'biliary tract tumor'/exp | 74131 |
| #2 | carcinoma*:ti,ab,kw OR cancer*:ti,ab,kw OR tumor*:ti,ab,kw OR neoplasm*:ti,ab,kw OR malign*:ti,ab,kw | 5660152 |
| #3 | cholangio*:ti,ab,kw OR 'bile duct':ti,ab,kw OR biliary:ti,ab,kw OR gallbladder:ti,ab,kw OR ampullary:ti,ab,kw | 263155 |
| #4 | #2 AND #3 | 88976 |
| #5 | #1 OR #4 | 115203 |
| #6 | 'immune checkpoint inhibitor'/exp OR 'chemotherapy'/exp OR 'immunotherapy'/exp OR 'molecularly targeted therapy'/exp | 1221901 |
| #7 | checkpoint inhibitors, immune':ti,ab,kw OR 'immune checkpoint blockers':ti,ab,kw OR 'checkpoint blockers, immune':ti,ab,kw OR 'immune checkpoint inhibitor':ti,ab,kw OR 'checkpoint inhibitor, immune':ti,ab,kw OR 'immune checkpoint blockade':ti,ab,kw  OR 'checkpoint blockade, immune':ti,ab,kw OR 'immune checkpoint inhibition':ti,ab,kw OR 'checkpoint inhibition, immune':ti,ab,kw | 30586 |
| #8 | drug therapy':ti,ab,kw OR 'chemo therapy':ti,ab,kw OR chemotherapies:ti,ab,kw OR pharmacotherapy:ti,ab,kw OR pharmacotherapies:ti,ab,kw  OR 'therapy, drug':ti,ab,kw OR 'drug therapies':ti,ab,kw OR 'therapies, drug':ti,ab,kw | 161237 |
| #9 | immunotherapies:ti,ab,kw OR 'molecular targeted therapies':ti,ab,kw OR 'targeted therapy, molecular':ti,ab,kw OR 'therapy, molecular targeted':ti,ab,kw  OR 'targeted molecular therapy':ti,ab,kw OR 'molecular therapy, targeted':ti,ab,kw OR 'targeted molecular therapies':ti,ab,kw OR 'therapy, targeted molecular':ti,ab,kw | 30986 |
| #10 | #6 OR #7 OR #8 OR #9 | 1379115 |
| #11 | randomized controlled trial':ti,ab,kw OR 'controlled clinical trial':ti,ab,kw OR randomized:ti,ab,kw OR randomised:ti,ab,kw OR  randomly:ti,ab,kw OR trial:ti,ab,kw OR phase:ti,ab,kw | 3575716 |
| #12 | first line':ti,ab,kw OR untreated:ti,ab,kw OR treatmentnaive:ti,ab,kw OR 'treatment naive':ti,ab,kw OR treatment:ti,ab,kw OR therapy:ti,ab,kw | 9804884 |
| #13 | #5 AND #10 AND #11 AND #12 | 2721 |

**(C) Cochrone library**

| ID | Search | Results |
| --- | --- | --- |
| #1 | MeSH descriptor: [Biliary Tract Neoplasms] explode all trees | 784 |
| #2 | MeSH descriptor: [Cholangiocarcinoma] explode all trees | 366 |
| #3 | MeSH descriptor: [Bile Duct Neoplasms] explode all trees | 415 |
| #4 | MeSH descriptor: [Gallbladder Neoplasms] explode all trees | 151 |
| #5 | (cholangio*):ti,ab,kw OR (biliary):ti,ab,kw OR (bile duct):ti,ab,kw OR (gallbladder):ti,ab,kw OR (ampullary):ti,ab,kw | 13029 |
| #6 | (carcinoma*):ti,ab,kw OR (tumor*):ti,ab,kw OR (neoplasm*):ti,ab,kw OR (cancer*):ti,ab,kw OR (malign*):ti,ab,kw | 281505 |
| #7 | #5 and #6 | 4145 |
| #8 | #1 OR #2 OR #3 OR #4 OR #7 | 4177 |
| #9 | MeSH descriptor: [Immune Checkpoint Inhibitors] explode all trees | 287 |
| #10 | (Checkpoint Inhibitors, Immune):ti,ab,kw OR (Immune Checkpoint Blockers):ti,ab,kw OR (Checkpoint Blockers, Immune):ti,ab,kw  OR (Immune Checkpoint Inhibitor):ti,ab,kw OR (Checkpoint Inhibitor, Immune):ti,ab,kw OR (Immune Checkpoint Blockade):ti,ab,kw  OR (Checkpoint Blockade, Immune):ti,ab,kw OR (Immune Checkpoint Inhibition):ti,ab,kw OR (Checkpoint Inhibition, Immune):ti,ab,kw | 2645 |
| #11 | MeSH descriptor: [Drug Therapy] explode all trees | 186057 |
| #12 | (Chemotherapy):ti,ab,kw OR (chemo-therapy):ti,ab,kw OR (Chemotherapies):ti,ab,kw OR (Pharmacotherapy):ti,ab,kw OR (Pharmacotherapies):ti,ab,kw OR (Therapy, Drug):ti,ab,kw OR (Drug Therapies):ti,ab,kw OR (Therapies, Drug):ti,ab,kw | 605995 |
| #13 | MeSH descriptor: [Immunotherapy] explode all trees | 12235 |
| #14 | (immunotherapies):ti,ab,kw | 536 |
| #15 | MeSH descriptor: [Molecular Targeted Therapy] explode all trees | 335 |
| #16 | (Targeted Molecular Therapy):ti,ab,kw OR (Molecular Therapy, Targeted):ti,ab,kw OR (Targeted Molecular Therapies):ti,ab,kw OR (Therapy, Targeted Molecular):ti,ab,kw | 1494 |
| #17 | #9 OR #10 OR #11 OR #12 OR #13 OR #14 OR #15 OR #16 | 656209 |
| #18 | (Randomized Controlled Trial):ti,ab,kw OR (controlled clinical trial):ti,ab,kw OR (randomized):ti,ab,kw OR (randomised):ti,ab,kw  OR (randomly):ti,ab,kw OR (trial):ti,ab,kw OR (phase):ti,ab,kw | 1505813 |
| #19 | (first line):ti,ab,kw OR (first-line):ti,ab,kw OR (untreated):ti,ab,kw OR (treatmentnaive):ti,ab,kw OR (treatment-naïve):ti,ab,kw  OR (treatment):ti,ab,kw OR (therapy):ti,ab,kw | 1287642 |
| #20 | #8 and #17 and #18 and #19 | 1452 |

| Targets | Drugs |
| --- | --- |
| MEK1/2 | Selumetinib |
| PD-1/PD-L1 | Pembrolizumab, Durvalumab, Sintilimab, Durvalumab |
| VEGFR | Ramucirumab, Cediranib |
| MET | Merestinib |
| Multi targets | Bintrafusp Alfa, Regorafenib, Anlotinib, Sorafenib, Vandetanib |
| EGFR | Cetuximab, Erlotinib, Panitumumab |
| CTLA-4 | Tremelimumab |
| TCA cycle | Devimistat (CPI-613） |
| FGFR | Infigratinib |

**Supplement S2: The drugs and their targets mentioned in the studies.**

**Supplement S3:** **Tumor sites in average group**

| **Study** | **Cholangiocarcinoma** | | **Gallbladder cancer** | **Ampulla cancer** | **Multifocal** | **unspecified** | **Periamp** | **Total** |
| --- | --- | --- | --- | --- | --- | --- | --- | --- |
|  | **Intrahepatic** | **Extrahepatic** |  |  |  |  |  |  |
| **Others** | 1767 | 637 | 670 | 30 | 2 | 303 | 15 | 3424 |
| **J. Lee 2012** | 180 | | 82 | 6 | 0 | 0 | 0 | 268 |
| **Total** | 2584 | | 752 | 36 | 2 | 303 | 15 | 3692 |

Periamp: common bile duct and ampulla cancer

**Supplement S4: Network evidence diagram**


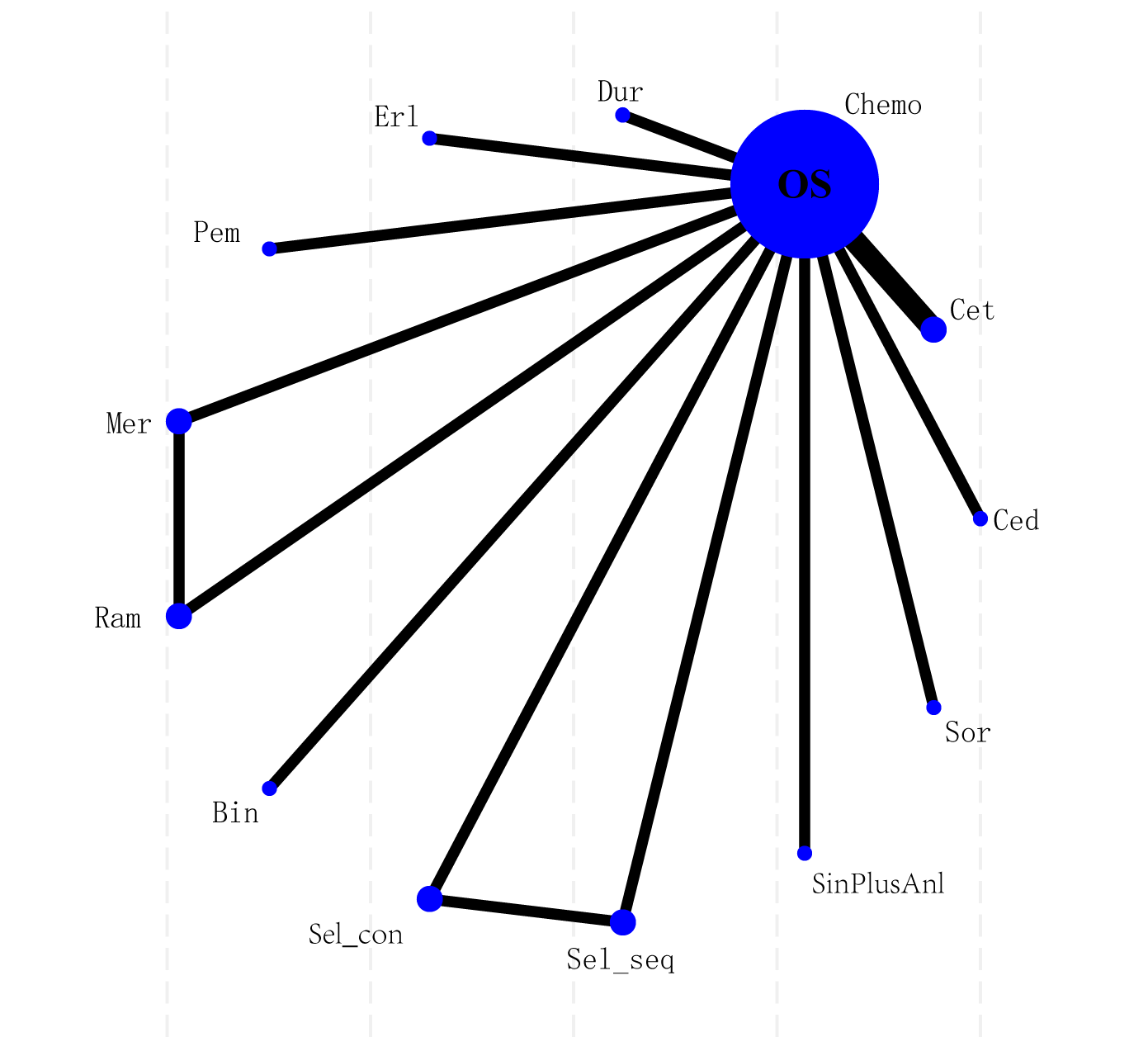

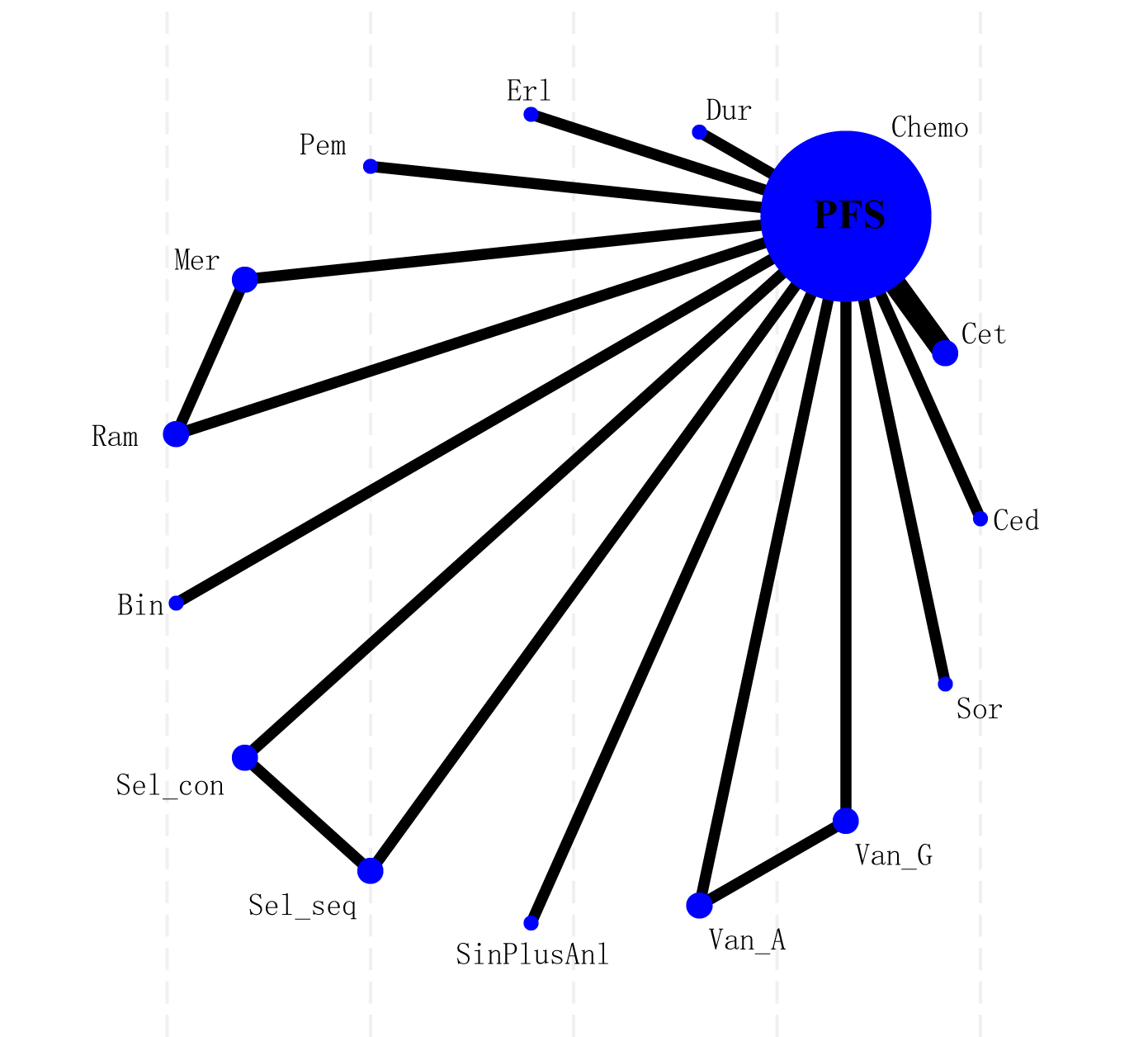

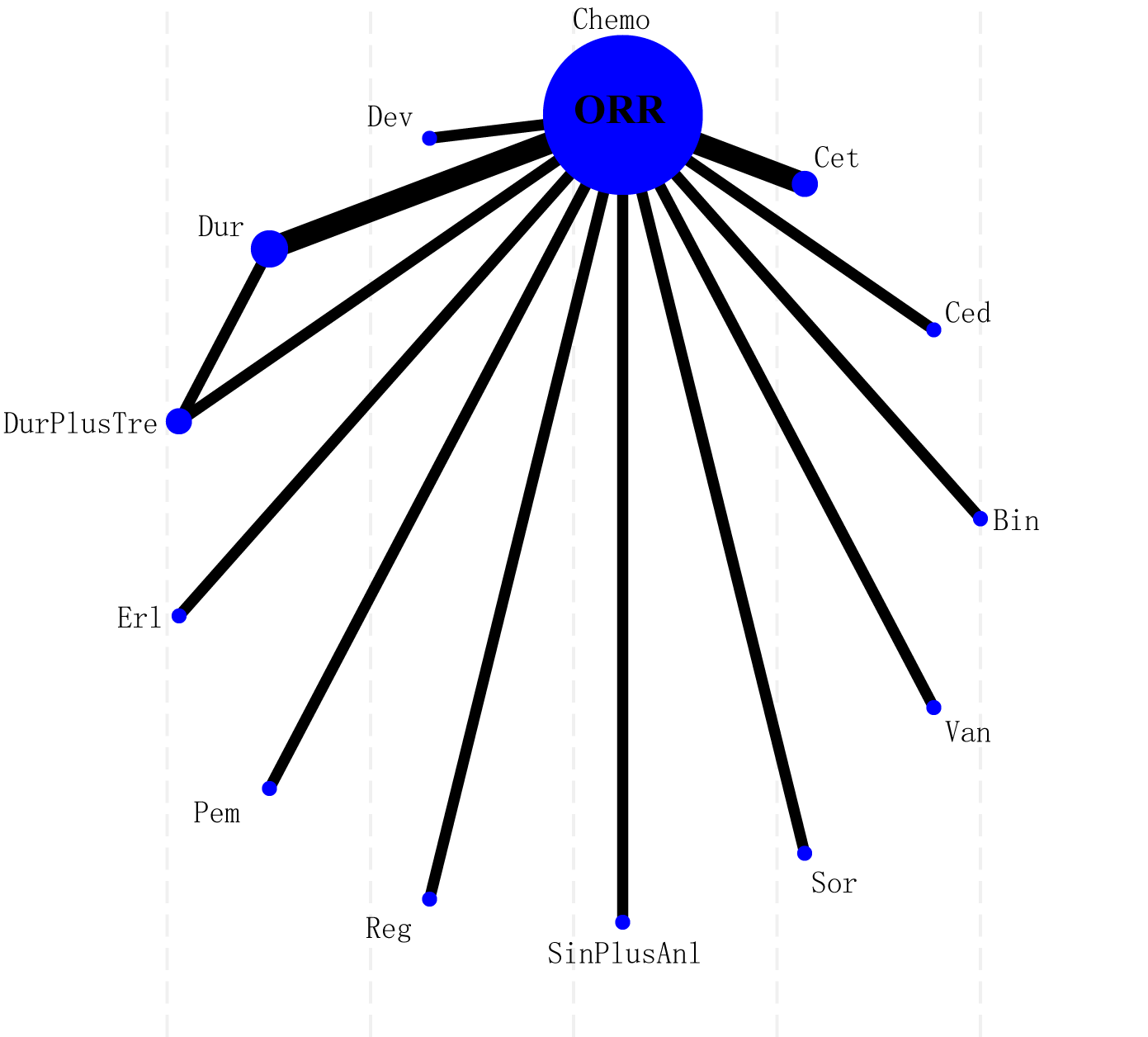

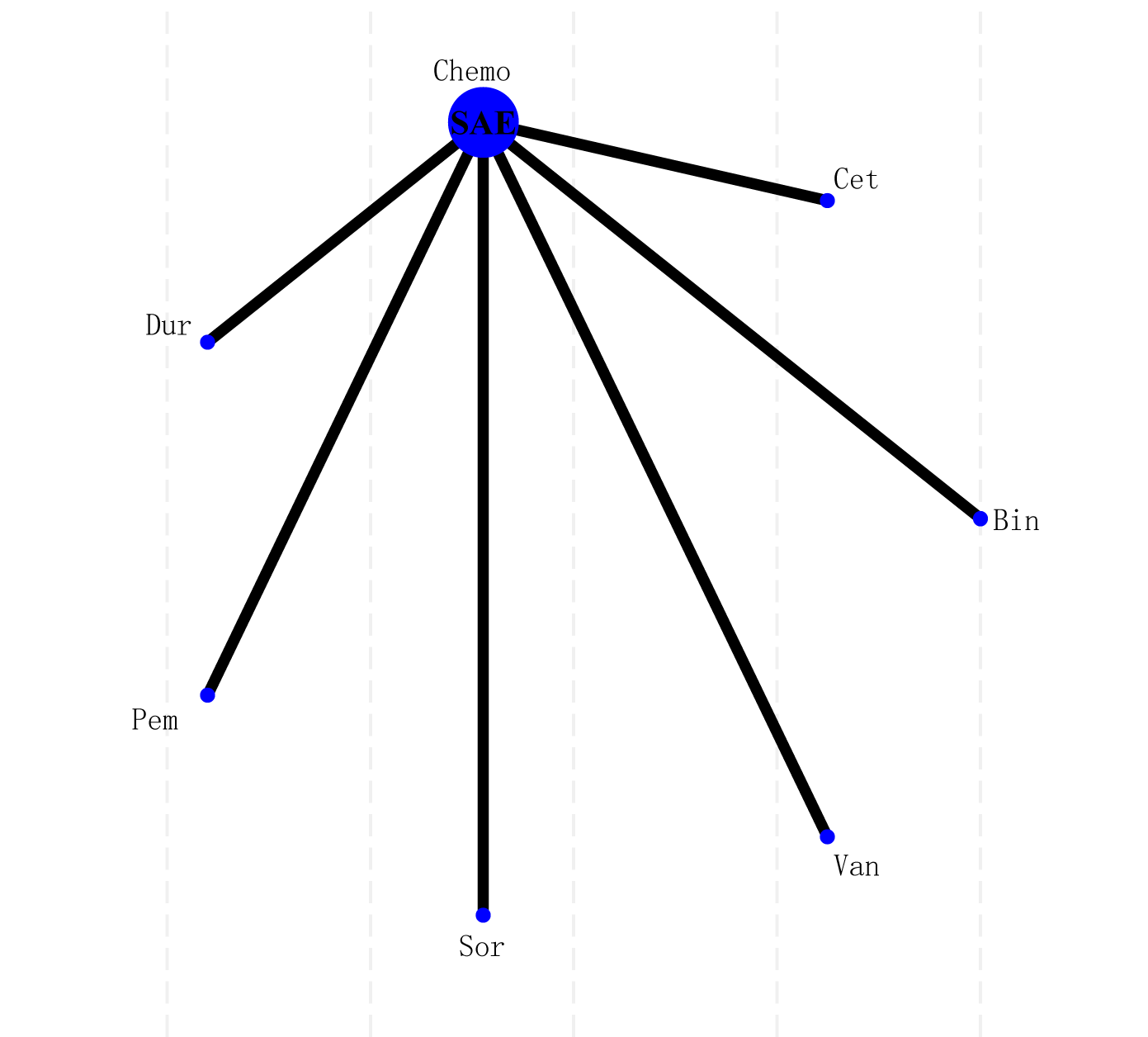


**Supplement S5: Heat map of SAE**

**
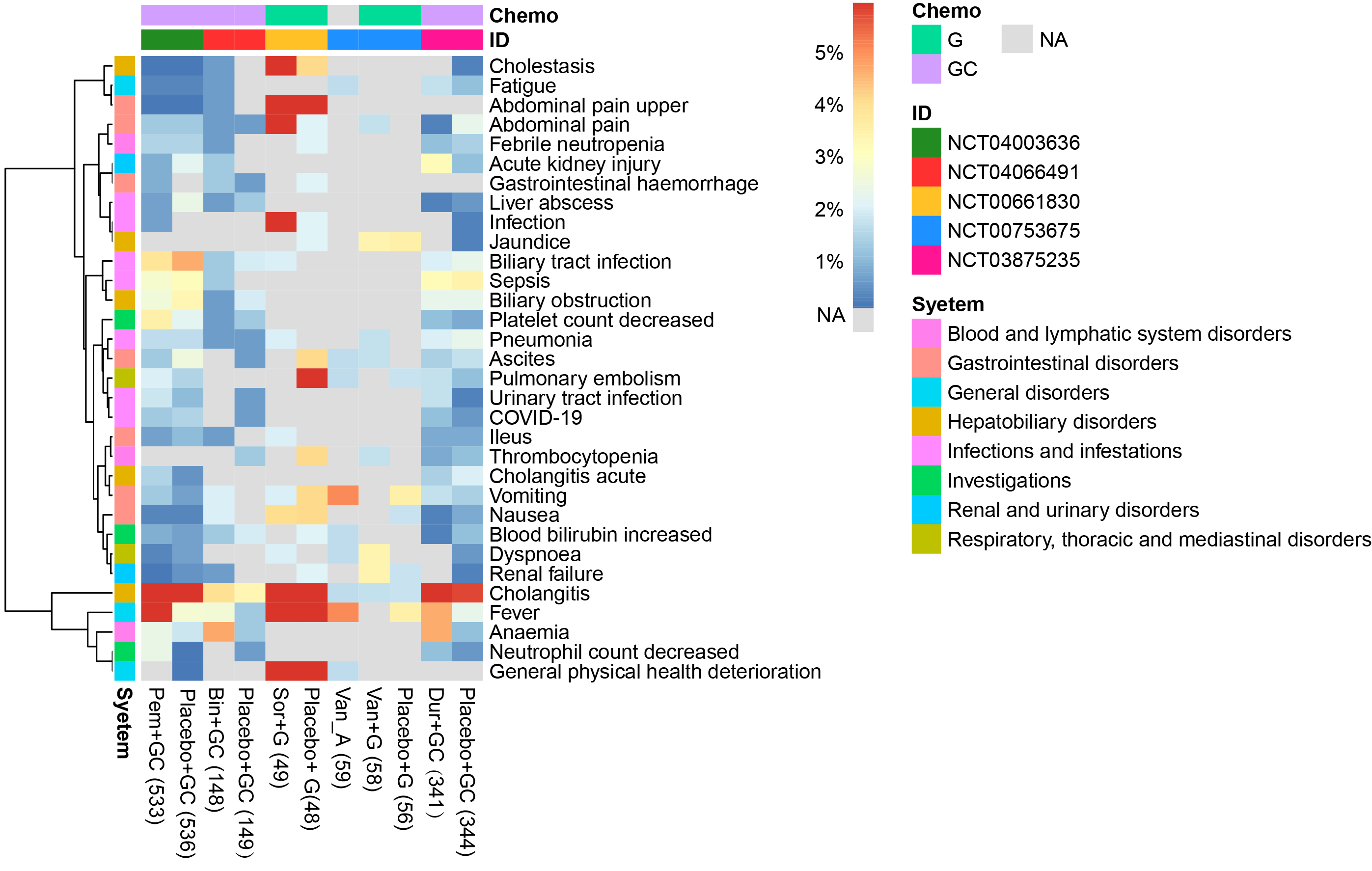
**

**Supplement S6: Risk of bias assessment**

**(A) Risk of bias assessment plots**

**
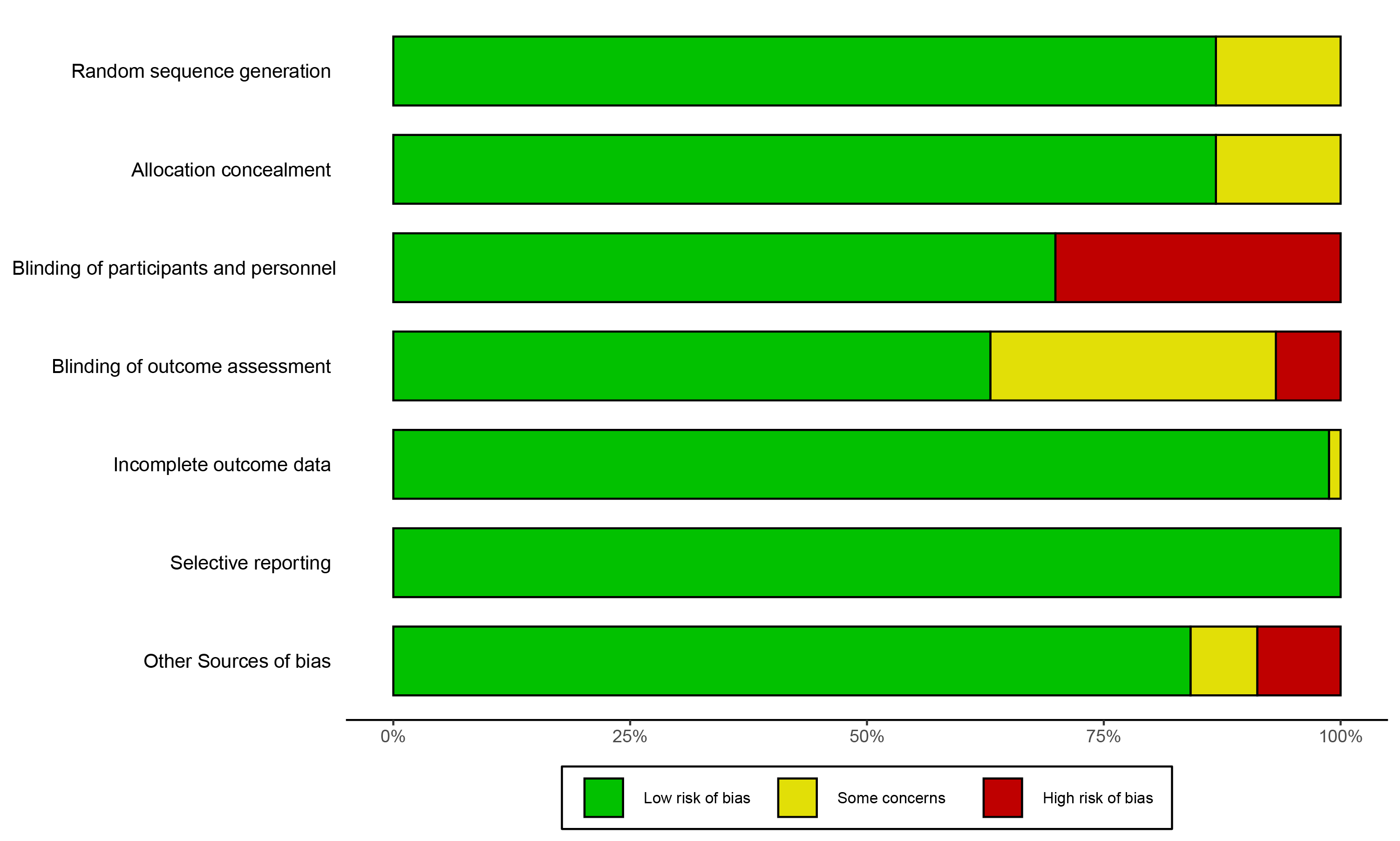
(B) Risk of bias proportion**

**(C) Detailed assessment of each study**

| Study | Random sequence generation | Allocation concealment | Blinding of participants and personnel | Blinding of outcome assessment | Incomplete outcome data | Selective reporting | Other Sources of bias |
| --- | --- | --- | --- | --- | --- | --- | --- |
| J. Li 2024 | Unclear, Description includes only 'randomization' | Unclear, No specific description | High, Open Label | Unclear, No specific description | Low, OS, PFS, ORR and AE reported number close to total random patients in each arm | Low, Results of all primary outcome measures were reported | Low, Baseline characteristics were well balanced |
| V. Sahai 2024 | Low, Randomization with Bayesian design Control Arm | Low, Bayesian | High, Open Label | Unclear, No specific description | Low, OS, PFS and ORR reported number close to total random patients in each arm | Low, Results of all primary outcome measures were reported | Unclear, No specific description |
| D.Y. Oh 2024 | Low, An interactive response system | Low, An interactive response system | Low, Double blind | Low, An Independent Data Monitoring Committee (IDMC) | Low, OS, PFS, ORR and AE reported number close to total random patients in each arm | Low, Results of all primary outcome measures were reported | High, Early terminated |
| R.K. Kelley 2023 | Low, An interactive voice-response system and a randomisation list | Low, An interactive voice-response system | Low, Double blind | Low, Data reviewed by independent committ | Low, OS, PFS, ORR and AE reported number close to total random patients in each arm | Low, Results of all primary outcome measures were reported | Low, Baseline characteristics were well balanced |
| D.Y. Oh 2024 | Low, A computer generated randomisation scheme | Low, An interactive voice response system or interactive web response system | Low, Double blind | Low, Masking: Quadruple (Participant, Care Provider,  Investigator, Outcomes Assessor) | Low, OS, PFS, ORR and AE reported number close to total random patients in each arm | Low, Results of all primary outcome measures were reported | Low, Baseline characteristics were well balanced |
| G.K. Abou-Alfa 2022 | Unclear, Description includes only 'random' | Unclear, No specific description | High, Open Label | Unclear, No specific description | Unclear, No specific description | Low, Results of all primary outcome measures were reported | High, Early terminated |
| A. Vogel 2022 | Unclear, Description includes only 'randomized' | Unclear, No specific description | High, Open Label | Unclear, No specific description | Low, OS, PFS, ORR and AE reported number close to total random patients in each arm | Low, Results of all primary outcome measures were reported | Unclear, No specific description |
| J.W. Valle 2021 | Low, A permuted block randomisation method | Low, an interactive web response system. | Low, Double blind | Unclear, No specific description | Low, OS, PFS, ORR and AE reported number close to total random patients in each arm | Low, Results of all primary outcome measures were reported | Low, Baseline characteristics were well balanced |
| E. Assenat 2021 | Unclear, Description includes only 'random' | Unclear, No specific description | High, Open Label | Unclear, No specific description | Low, OS, PFS, ORR and AE reported number close to total random patients in each arm | Low, Results of all primary outcome measures were reported | Unclear, No specific description |
| Mark K. Doherty 2022 | Low, An interactive voice recognition system | Low, An interactive voice recognition system | High, Open Label | Unclear, No specific description | Low, OS, PFS and AE reported number close to total random patients in each arm | Low, Results of all primary outcome measures were reported | Low, Baseline characteristics were well balanced |
| F. Leone 2015 | Low, A computed system with permuted-block randomization | Low, Computed system | High, Open Label | Unclear, No specific description | Low, OS, PFS, ORR and AE reported number close to total random patients in each arm | Low, Results of all primary outcome measures were reported | Low, Baseline characteristics were well balanced |
| A. Vogel 2018 | Unclear, Description includes only 'randomized' | Unclear, No specific description | High, Open Label | Unclear, No specific description | Low, OS, PFS and AE reported number close to total random patients in each arm | Low, Results of all primary outcome measures were reported | Low, Baseline characteristics were well balanced |
| J.S. Chen 2015 | Low, A permutated block randomization | Low, Carried out centrally at the Statistical Center of TCOG | High, Open Label | Low, The evaluator was blind to the treatment received by patients | Low, OS, PFS, ORR and AE reported number close to total random patients in each arm | Low, Results of all primary outcome measures were reported | Low, Baseline characteristics were well balanced |
| J. Lee 2012 | Low, A permutated block randomization | Low, Randomisation Generator (version 1.0) software was used | High, Open Label | High, Investigators who assessed the response to the treatment were not masked to group assignment | Low, OS, PFS, ORR and AE reported number close to total random patients in each arm | Low, Results of all primary outcome measures were reported | Low, Baseline characteristics were well balanced |
| J.W. Valle 2015 | Low, Computer with a minimisation algorithm | Low, An interactive web-based response system | Low, Double blind | Low, Masking: Quadruple (Participant, Care Provider,  Investigator, Outcomes Assessor) | Low, OS, PFS, ORR and AE reported number close to total random patients in each arm | Low, Results of all primary outcome measures were reported | Low, Baseline characteristics were well balanced |
| A. Santoro 2015 | Low, A computer-generated randomization scheme (stratified by center) | Low, sequentially numbered opaque, sealed envelopes. | Low, Double blind | Low, Masking: Triple (Participant, Investigator, Outcomes Assessor) | Low, OS, PFS, ORR and AE reported number close to total random patients in each arm | Low, Results of all primary outcome measures were reported | Low, Baseline characteristics were well balanced |
| D. Malka 2014 | Low, A minimisation procedure | Low, By the institut Gustave Roussy Biostatistics Unit | High, Open Label | Unclear, No specific description | Low, OS, PFS, ORR and AE reported number close to total random patients in each arm | Low, Results of all primary outcome measures were reported | Low, Baseline characteristics were well balanced |
| M. Moehler 2014 | Unclear, Description includes only 'randomized' | Unclear, No specific description | Low, Double blind | Unclear, No specific description | Low, OS, PFS and AE reported number close to total random patients in each arm | Low, Results of all primary outcome measures were reported | Low, Baseline characteristics were well balanced |

**Supplement S7: I2 and DIC differences**

| **Average group** | **PFS** | **OS** | **ORR** | **SAE** |
| --- | --- | --- | --- | --- |
| **I^2^** | 16% | 2% | 10% | 9% |
| **DIC consistency** | 30.67532 | 24.1805 | 54.3475 | 24.0997 |
| **DIC inconsistency** | 30.64591 | 24.1531 | 54.2499 | 24.1061 |
| **Difference** | 0.02941 | 0.0274 | 0.0976 | 0.0064 |

**Supplement S8: Funnel plots**

1. **
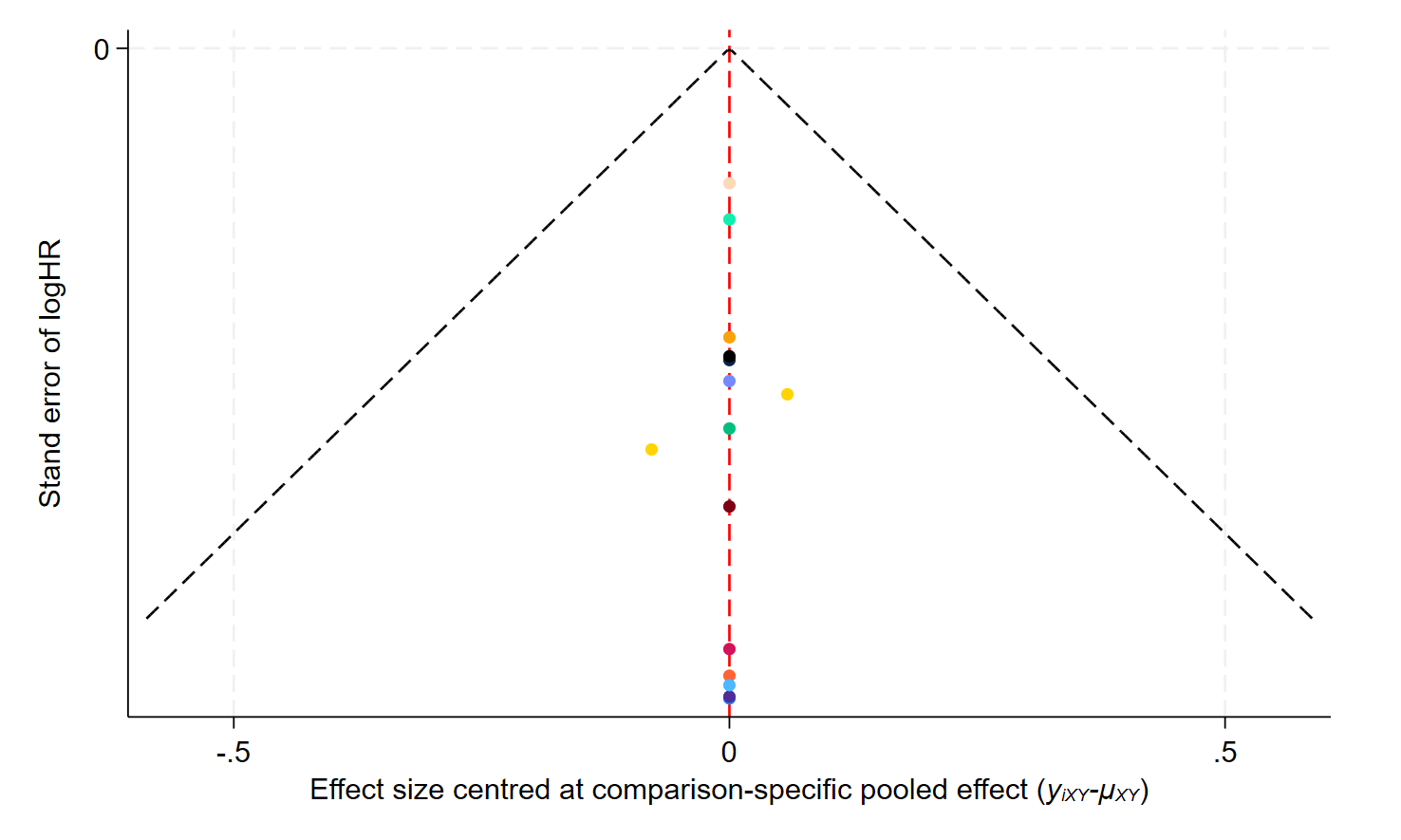
 The funnel plots of OS**
2. **The funnel plots of PFS**
3. **
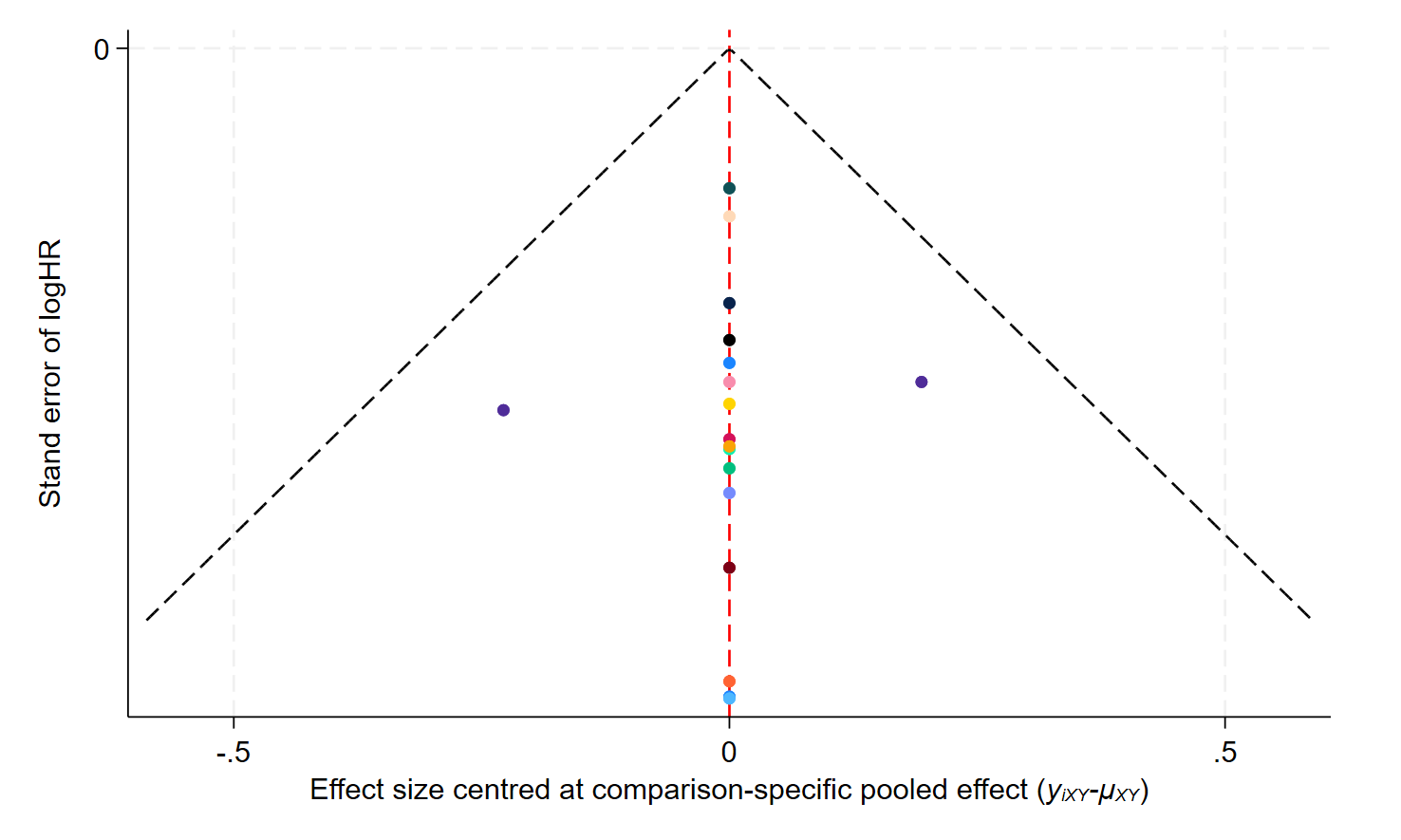
 The funnel plots of ORR**
4. **
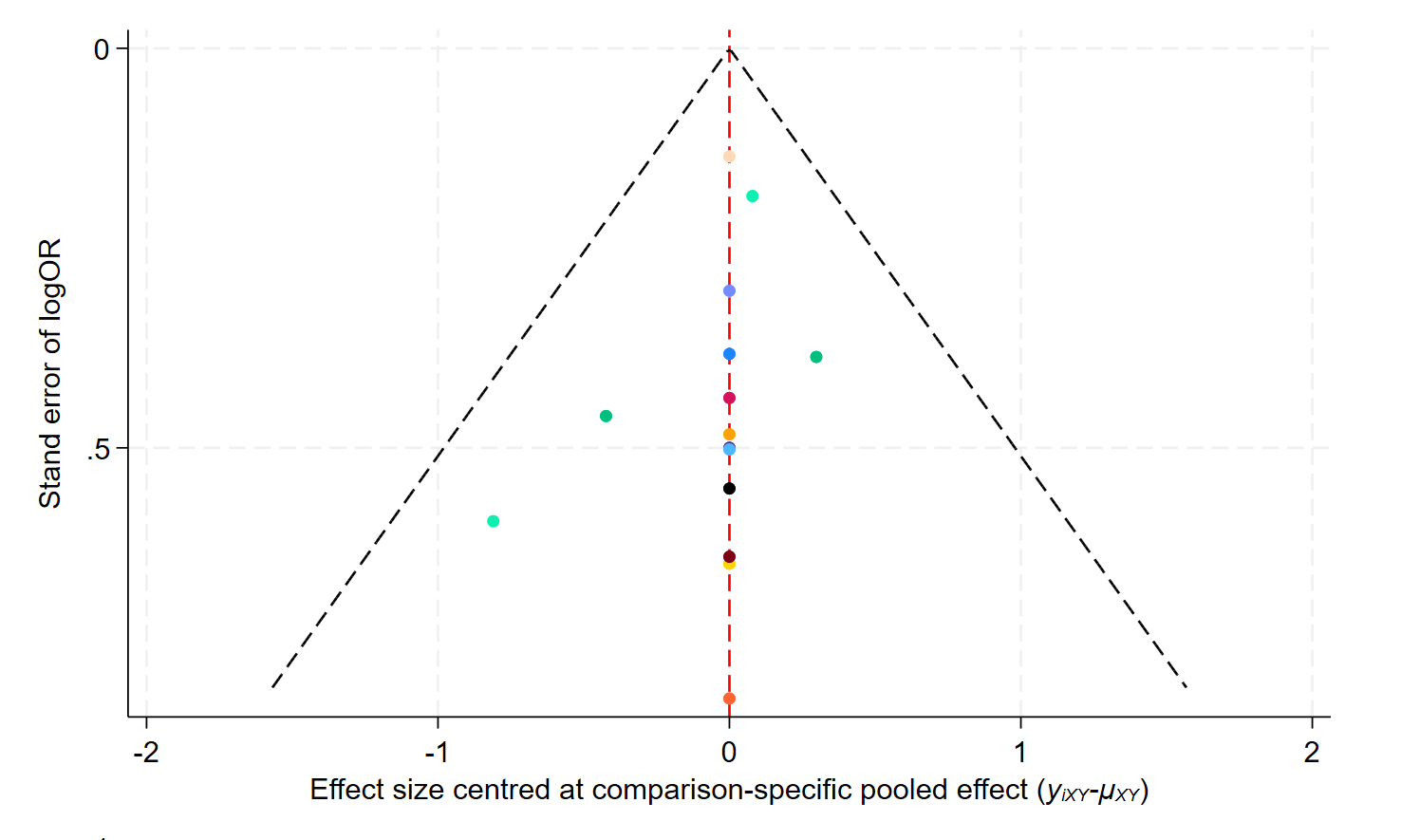
The funnel plots of SAE**

**
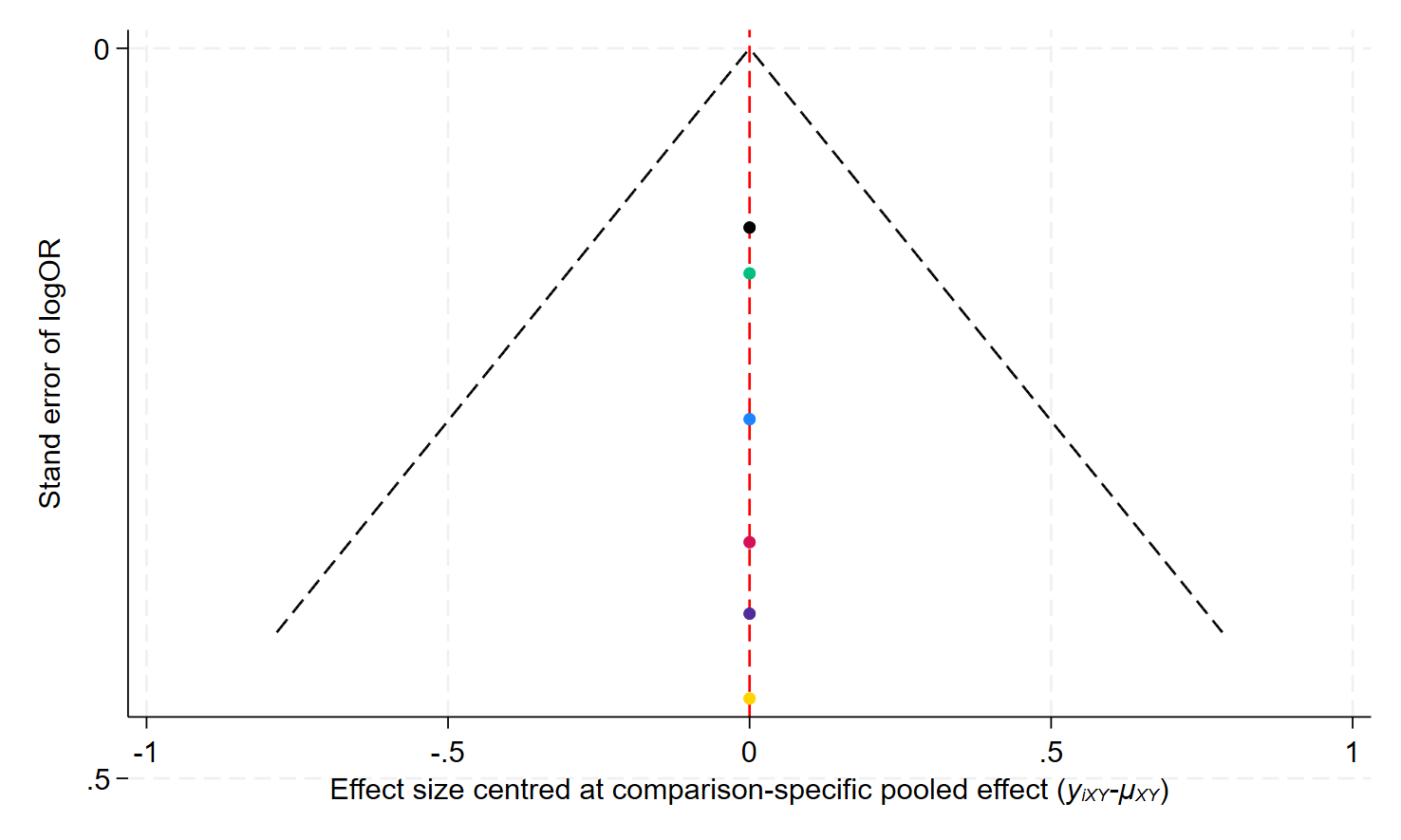
**

| **Supplement S9: Basic features of included studies** | | | | | | | | | | | | |
| --- | --- | --- | --- | --- | --- | --- | --- | --- | --- | --- | --- | --- |
| **Study** | **Journal of publication** | **Age （median）** | **Region** | **Tumor sites** | **Advanced situation** | **size** | **Treatment arms** | **OS-HR**  **(95% CI)** | **PFS-HR**  **(95% CI)** | **ORR** | **SAE** | **Subgroup** |
| J. Li 2024^1^ NCT04300959 RCT II | Nature Portfolio | A:62.5 B:59.5 | China | IHC 50  EHC 11 GBC 19 | Locally Advanced Metastases Recurrent | 80 | A: sintilimab plus anlotinib + GC (40)  B: GC (40) | 1.04 (0.40-1.49) | 0.47 (0.22-0.64) | A:19/37 B:10/34 | NA |  |
| V. Sahai 2024^2^ NCT04203160 RCT Ⅰ/Ⅱ | Journal of Clinical Oncology | NA | USA | IHC 48 EHC 13 GBC 7 | Locally Advanced Metastatic | 75 | A：Devimistat + GC (57) B：GC (18) | NA | NA | A:10/34 B:6/13 | NA |  |
| D.Y. Oh 2024^3^ NCT04066491 RCT II/Ⅲ | Hepatology | A：64 B：65 | Multiple countries | IHC 132 EHC 66 GBC 78 APC 21 | Locally Advanced Metastatic | 297 | A: Bintrafusp alfa + GC (148) B：Placebo + GC (149) | 1.23 (0.66-2.28) | 1.08 (0.70–1.66) | A:23/73 B:15/77 | A:58/146  B:36/149 |  |
| R.K. Kelley 2023^4^ NCT04003636 RCT Ⅲ | The Lancet Oncology | A:64 B:63 | Multiple countries | IHC 633 EHC 203 GBC 233 | Locally Advanced Metastatic | 1069 | A: Pembrolizumab + GC (533) B: Placebo + GC (536) | 0.83 (0.72-0.95) | 0.86 (0.75-1.00) | A:153/533 B:153/536 | A:276/529 B:263/534 |  |
|  | The Lancet Oncology | NA | Multiple countries | NA | NA | 728 | A: Pembrolizumab + GC (363) B: Placebo + GC (365) | 0.85 (0.72-1.00) | NA | NA | NA | PD-L1 expression ≥1% |
|  | The Lancet Oncology | NA | Multiple countries | NA | NA | 223 | A: Pembrolizumab + GC (113) B: Placebo + GC (110) | 0.84  (0.62–1.14) | NA | NA | NA | PD-L1 expression <1% |
| D.Y. Oh 2024^5^ NCT03875235 RCT Ⅲ | The Lancet Gastroenterology and Hepatology | A:64 B:64 | Multiple countries | IHC 383 EHC 131 GBC 171 | Locally Advanced Metastatic Recurrent | 685 | A: Durvalumab + GC (341) B: Placebo + GC (344) | 0·76 (0.64-0.91) | 0.75 (0.63-0.89) | A:91/341 B:64/343 | A:160/338 B:149/342 |  |
|  | The Lancet Gastroenterology and Hepatology | NA | Multiple countries | NA | NA | 406 | A: Durvalumab + GC (199) B: Placebo + GC (207) | 0.75 (0.60-0.93) | NA | NA | NA | PD-L1 expression ≥1% |
|  | The Lancet Gastroenterology and Hepatology | NA | Multiple countries | NA | NA | 206 | A：Durvalumab + GC (103) B：Placebo + GC (103) | 0.79 (0.58-1.09) | NA | NA | NA | PD-L1 expression <1% |
| A. Vogel 2022^6^ NCT03473574 RCT II | Annals of Oncology | NA | Germany | IHC 98 EHC 27  GBC 13 | Metastatic | 138 | A: Durvalumab plus Tremelimumab + G/GC (22/52) B: GC (35) C: Durvalumab + GC (29) | NA | NA | A:13/74 B:10/35 C:6/29 | NA |  |
| J.W. Valle 2021^7^ NCT02711553 RCT II | The Lancet Oncology | NA | Multiple countries | IHC EHC GBC | Locally Advanced Metastatic Recurrent | 294 | A: Ramucirumab + GC (103) B: Merestinib + GC (95) C: Pooled Placebo + GC (96) | 1.42  (1.03-1.96) 0.97 (0.69-1.37) | 1.08 (0.78-1.49) 0.88 (0.63-1.25) | NA | NA |  |
| E. Assenat 2021^8^ NCT02386397 RCT II | Annals of Oncology | NA | France | IHC 39 EHC 13  GBC 11 | Locally Advanced Metastatic Recurrent | 63 | A：Regorafenib + GO (42) B：GO (21) | NA | NA | A:14/42 B:4/21 | NA |  |
| Mark K. Doherty 2022^9^ NCT02151084 RCT II | British Journal of Cancer | A:60 B:61 C:64 | Canada | IHC 22 EHC 16 GBC 19 | Locally Advanced Metastatic | 57 | A: continuous selumetinib + GC (n=19) B: sequential selumetinib + GC (n=19) C: GC (n=19) | 0.96 (0.49-1.88) 1.26 (0.66-2.40)^🌢^ | 1.02 (0.53-1.95) 0.84 (0.43-1.64)^🌢^ | NA | NA |  |
| J.S. Chen 2015^10^ NCT01267344 RCT II | Annals of Oncology | A:61 B:59 | China | IHC 89 EHC 19 GBC 14 | Locally Advanced Metastatic | 122 | A: cetuximab + GO (62) B: GO(60) | 0.99 (0.66-1.50)^🌢^ | 0.70 (0.48-1.01) | A:17/62 B:9/60 | NA |  |
|  | Annals of Oncology | NA | China | NA | NA | 45 | A: Cetuximab + GO (17) B: GO (28) | NA | 0.72 (0.38-1.34) | A:6/17 B:4/28 | NA | EGFR wild-type |
|  | Annals of Oncology | NA | China | NA | NA | 75 | A: Cetuximab + GO (43) B: GO (32) | NA | 0.62 (0.38-1.00) | A:10/43 B: 5/32 | NA | EGFR mutation |
|  | Annals of Oncology | NA | China | NA | NA | 78 | A: Cetuximab + GO (39) B: GO (39) | NA | 0.65 (0.41-1.03) | A:12/39 B: 6/39 | NA | KRAS wild-type |
|  | Annals of Oncology | NA | China | NA | NA | 44 | A: Cetuximab + GO (23) B: GO (21) | NA | 0.73 (0.39-1.35) | A:5/23 B:3/21 | NA | KRAS mutation |
| J. Lee 2012^11^ NCT01149122 RCT Ⅲ | The Lancet Oncology | A:59 B:61 | Korea | IHC/EHC 180 GBC 82  APC 6 | Metastatic Recurrent | 268 | A：erlotinib + GO (n=135) B：GO (n=133) | 0.93 (0.69-1.25) | 0·80 (0.61-1.03) | A:40/135 B:21/133 | NA |  |
| J.W. Valle 2015^12^ NCT00939848 RCT II | The Lancet Oncology | A:68 B:64.5 | United Kingdom | IHC 29 EHC 48  GBC 39 APC 8 | Locally Advanced Metastatic Recurrent | 124 | A：cediranib + GC (62) B: Placebo+GC (62) | 0.86 (0.58-1.27) | 0.93 (0.65-1.35) | A:26/59 B:10/54 | NA |  |
| A. Santoro 2015^13^ NCT00753675 RCT II | Annals of Oncology | NA | Italy | IHC 87 EHC 39  GBC 31  unspecified 1 Periamp 15 | Locally Advanced Metastatic | 173 | A: Vandetanib (59) B: Vandetanib+ G (58) C: Placebo+G (56) | NA | 1.30 (0.86-1.96) 1.30 (0.75-1.70) | A:2/56 B:11/57 C:7/52 | A:16/59 B:15/58 C:12/56 |  |
| D. Malka 2014^14^ NCT00552149 RCT II | The Lancet Oncology | A:61 B:62 | France | IHC 95 EHC 22 GBC 22  APC 1 Multifocal 2 unspecified 8 | Locally Advanced Metastatic | 150 | A: cetuximab + GO (n=76) B: GO (n=74) | 1.14 (0.80-1.63)^🌢^ | 1.07 (0.76-1.50)^🌢^ | A:18/76 B:17/74 | A:39/76 B:25/71 |  |
| M. Moehler 2014^15^ NCT00661830 RCT II | European Journal of Cancer | A：64 B：64.5 | Germany | IHC 62  EHC 22 GBC 13 | Locally Advanced Metastatic | 97 | A: Sorafenib + G (49) B: Placebo + G (48) | 1.20 (0.75-1.93) | 1.28 (0.81-2.02) | A:4/28 B:3/30 | A：33/49 B：35/48 |  |
| A. Vogel 2018^16^ NCT01320254 RCT II | European Journal of Cancer | A:62 B:59.5 | Germany | IHC 61 EHC 24 GBC 14 | Locally Advanced Metastatic | 90 | A: Panitumumab + GC (62) B: GC (28) | 1.43 (0.85-2.44) | 1.37 (0.83-2.22) | NA | NA | KRAS wild-type |
| F. Leone 2015^17^ NCT01389414 RCT II | Cancer | NA | Italy | IHC 42 EHC 19 GBC 28 | Locally Advanced Metastatic | 89 | A: Panitumumab + GO (45) B: GO (44) | 0.83 (0.53-1.3) | 0.78 (0.51-1.21) | A:12/45 B: 8/44 | A: 18/45 B: 12/44 | KRAS wild-type |
| G.K. Abou-Alfa 2022 NCT03773302^18^ RCT Ⅲ | Journal of Clinical Oncology | NA | Multiple countries | IHC 45 EHC 3 | Locally Advanced Metastatic | 48 | A：Infigratinib （29） B：GC (19) | NA | NA | A:11/29 B：3/19 | A:10/29  B：0/17 | FGFR mutation |

**References：**

1. Li, J. *et al.* Phase Ⅱ Study of Combined Sintilimab and Anlotinib with Gemcitabine plus Cisplatin in Advanced Biliary Tract Cancer: Efficacy, Safety and Optimize Dose. Preprint at https://doi.org/10.21203/rs.3.rs-4557891/v1 (2024).

2. Sahai, V. *et al.* Phase 1b/2 results of a multicenter, randomized phase 1b/2 study of gemcitabine and cisplatin +/- devimistat as first-line therapy for patients with advanced biliary tract cancer (BilT-04). *JCO* **42**, 4116–4116 (2024).

3. Oh, D.-Y. *et al.* Bintrafusp alfa and chemotherapy as first-line treatment in biliary tract cancer: A randomized phase 2/3 trial. *Hepatology* (2024) doi:10.1097/HEP.0000000000000965.

4. Kelley, R. K. *et al.* Pembrolizumab in combination with gemcitabine and cisplatin compared with gemcitabine and cisplatin alone for patients with advanced biliary tract cancer (KEYNOTE-966): a randomised, double-blind, placebo-controlled, phase 3 trial. *The Lancet* **401**, 1853–1865 (2023).

5. Oh, D.-Y. *et al.* Durvalumab or placebo plus gemcitabine and cisplatin in participants with advanced biliary tract cancer (TOPAZ-1): updated overall survival from a randomised phase 3 study. *The Lancet Gastroenterology & Hepatology* **9**, 694–704 (2024).

6. Vogel, A. *et al.* 52MO A randomized phase II trial of durvalumab and tremelIMUmab with gemcitabine or gemcitabine and cisplatin compared to gemcitabine and cisplatin in treatment-naïve patients with CHolangio- and gallbladdEr Carcinoma (IMMUCHEC). *Annals of Oncology* **33**, S563 (2022).

7. Valle, J. W. *et al.* Addition of ramucirumab or merestinib to standard first-line chemotherapy for locally advanced or metastatic biliary tract cancer: a randomised, double-blind, multicentre, phase 2 study. *The Lancet Oncology* **22**, 1468–1482 (2021).

8. Assenat, E. *et al.* 48P (BREGO) Regorafenib combined with modified m-GEMOX in patients with advanced biliary tract cancer (BTC): A phase II randomized trial. *Annals of Oncology* **32**, S376–S377 (2021).

9. Mark K. Doherty *et al.* Randomised, Phase II study of selumetinib, an oral inhibitor of MEK, in combination with cisplatin and gemcitabine chemotherapy for patients with advanced biliary tract cancer. *Br J Cancer* **127**, 1473–1478 (2022).

10. Chen, J. S. *et al.* A KRAS mutation status-stratified randomized phase II trial of gemcitabine and oxaliplatin alone or in combination with cetuximab in advanced biliary tract cancer. *Annals of Oncology* **26**, 943–949 (2015).

11. Lee, J. *et al.* Gemcitabine and oxaliplatin with or without erlotinib in advanced biliary-tract cancer: a multicentre, open-label, randomised, phase 3 study. *The Lancet Oncology* **13**, 181–188 (2012).

12. Valle, J. W. *et al.* Cediranib or placebo in combination with cisplatin and gemcitabine chemotherapy for patients with advanced biliary tract cancer (ABC-03): a randomised phase 2 trial. *The Lancet Oncology* **16**, 967–978 (2015).

13. Santoro, A. *et al.* A randomized, multicenter, phase II study of vandetanib monotherapy versus vandetanib in combination with gemcitabine versus gemcitabine plus placebo in subjects with advanced biliary tract cancer: the VanGogh study. *Annals of Oncology* **26**, 542–547 (2015).

14. Malka, D. *et al.* Gemcitabine and oxaliplatin with or without cetuximab in advanced biliary-tract cancer (BINGO): a randomised, open-label, non-comparative phase 2 trial. *The Lancet. Oncology* **15**, 819 (2014).

15. Moehler, M. *et al.* Gemcitabine plus sorafenib versus gemcitabine alone in advanced biliary tract cancer: A double-blind placebo-controlled multicentre phase II AIO study with biomarker and serum programme. *European Journal of Cancer* **50**, 3125–3135 (2014).

16. Vogel, A. *et al.* PICCA study: panitumumab in combination with cisplatin/gemcitabine chemotherapy in KRAS wild-type patients with biliary cancer—a randomised biomarker-driven clinical phase II AIO study. *European Journal of Cancer* **92**, 11–19 (2018).

17. Leone, F. *et al.* Panitumumab in combination with gemcitabine and oxaliplatin does not prolong survival in wild‐type KRAS advanced biliary tract cancer: A randomized phase 2 trial (Vecti‐BIL study). *Cancer* **122**, 574–581 (2015).

18. Abou-Alfa, G. K. *et al.* PROOF 301: A multicenter, open-label, randomized, phase 3 trial of infigratinib versus gemcitabine plus cisplatin in patients with advanced cholangiocarcinoma with an *FGFR2* gene fusion/rearrangement. *JCO* **40**, TPS4171–TPS4171 (2022).
